# Ever Use of Telehealth in Nebraska by March 2021: Cross-Sectional Analysis

**DOI:** 10.2196/53320

**Published:** 2024-11-28

**Authors:** Lisa C Smith, George Johnson, Snehal Jadhav, Josiane Kabayundo, Muskan Ahuja, Hongmei Wang, Kendra L Ratnapradipa

**Affiliations:** 1 Grace Abbott School of Social Work University of Nebraska Omaha Omaha, NE United States; 2 Department of Health Promotion University of Nebraska Medical Center Omaha, NE United States; 3 Department of Epidemiology University of Nebraska Medical Center Omaha, NE United States; 4 Department of Health Services Research & Administration University of Nebraska Medical Center Omaha, NE United States

**Keywords:** COVID-19, digital divide, health care access, telehealth, cross-sectional study, Nevada, United States, adult, medical care, geographical area, disparity, accessibility, utilization, survey, chi-square test, regression model, socioeconomic, demographic, health condition, digital health

## Abstract

**Background:**

Nationally, COVID-19 spurred the uptake of telehealth to facilitate patients’ access to medical care, especially among individuals living in geographically isolated areas. Despite the potential benefits of telehealth to address health care access barriers and enhance health outcomes, there are still disparities in the accessibility and utilization of telehealth services. Hence, identifying facilitators and barriers to telehealth should be prioritized to ensure that disparities are mitigated rather than exacerbated.

**Objective:**

This study aims to identify factors associated with ever use of telehealth in Nebraska, a primarily rural state with a significant portion of its population living in nonmetropolitan areas.

**Methods:**

A stratified random sample of Nebraska households (n=5300), with oversampling of census tracts with at least 30% African American, Hispanic, or Native American populations, received a mailed survey (English and Spanish) with web-based response options about social determinants of health and health care access (October 2020-March 2021). Survey weights were used for all calculations. Chi-square tests were used to compare telehealth use (yes or no) by participant sociodemographic, health, and access variables. Robust Poisson regression models were used to compute prevalence odds ratios (POR) with 95% CIs of telehealth use after controlling for socioeconomic, demographic, and health conditions.

**Results:**

The overall response rate was 20.8% (1101/5300). About 25.5% of Nebraska adults had ever used telehealth (urban 26.4%, rural 20.8%), despite 97% of respondents reporting internet access (98.3% urban, 90.5% rural). In the chi-square analysis, telehealth use was statistically significantly more common (*P*<.05) among those who are aged <45 years (32.4%), female (30.7%), and non-Hispanic (25.9%); with at least a bachelor’s degree (32.6%); who had a routine checkup (30.2%) or health care visit other than a routine checkup (34.2%); and with any chronic health conditions (29.6%) but did not differ (*P*≥.05) by race, marital status, income, insurance, having a primary care provider, or 1-way travel time for medical visits. In univariate models, internet access, age, sex, ethnicity, education, any health care visit in the past year, and no chronic health condition were significant (*P*<.05). When adjusted, education (POR 1.87, 95% CI 0.33-10.63) and sex (1.38, 0.93-2.04) were not significant, but internet access (5.43, 1.62-18.16), age <45 (5.33, 2.22-12.81) and 45-64 years (9.05, 2.37-34.62), non-Hispanic ethnicity (7.40, 2.39-22.90), any health care visit (2.43, 1.23-4.79), and any chronic condition (1.73, 1.09-2.76) were significantly associated with having ever used telehealth.

**Conclusions:**

This study highlights disparities in telehealth use. Despite high coverage, internet access was a significant predictor of telehealth use, highlighting the role of the digital divide in telehealth access and use. Telehealth use was significantly less prevalent among older adults, people without chronic health conditions, and Hispanic individuals. Targeted interventions that address barriers to telehealth use and improve health care access are warranted.

## Introduction

Telehealth, an umbrella term including telemedicine and nonclinical functions such as health education and patient support services, is a health care delivery model that uses technology to provide medical services remotely [1]. It encompasses a wide range of services and applications, all aimed at delivering health care consultations, monitoring, education, and support over digital communication channels [2]. Telehealth has gained significant prominence and acceptance, particularly in recent years, due to advances in technology and the need for more accessible and convenient health care options.

Telehealth has unquestionably widened health care access in the United States. Evidence shows it has effectively removed barriers associated with traveling to care [3]. It has been demonstrated to enhance health outcomes for individuals who are unable to physically visit health care providers but have access through phone or video platforms [2]. Additionally, there is compelling evidence indicating that the internet has empowered individuals to take charge of their health care utilization by accessing supplementary health information, managing personal health data, and engaging in meaningful communication with health care providers concerning their health conditions or enhancing disease management [4]. Telehealth has created avenues for patients to access information on disease management, receive medication advice, and be more actively engaged in their care.

In more recent years, telehealth has experienced a remarkable surge due to the COVID-19 pandemic [5]. The global health crisis forced health care systems and professionals to rapidly adapt to new methods of delivering care while prioritizing the safety of both patients and health care workers [5]. Telehealth emerged as a vital tool during this period, playing a pivotal role in the pandemic response [5]. It allowed medical professionals to assess and diagnose, provide treatment and follow-up, and monitor and track individuals while minimizing exposure [6]. While the surge in telehealth usage was initially driven by necessity, its value in providing accessible, efficient, and safe health care delivery is likely to endure in the post-pandemic health care landscape.

A persistent barrier to the expanding use of telehealth and its ability to address health disparities is the digital divide. The digital divide is the gap or disparity in access to and use of digital technologies, particularly the internet, between different groups of people or communities [7,8]. The digital divide encompasses access to internet connectivity, internet speed, the availability of digital devices, digital literacy, and the ability to effectively use digital tools for various purposes [9,10]. The digital divide is a significant social and economic issue, as it can have far-reaching consequences for individuals and communities [11].

Several characteristics have been linked to the widening of the digital divide, including age, educational attainment, income, sex, health status, racial or ethnic background, and geographic location [12]. The digital divide has had a significant impact on individuals, making them less likely to access health care–related information and potentially hindering their ability to comprehend information available through digital sources [13]. According to data from the Federal Communications Commission (FCC), which regulates interstate and international communications in the United States, there has been a notable disparity in broadband coverage across different regions, although it is gradually narrowing. Specifically, broadband coverage expanded in urban areas from 96.7% in 2015 to 98.8% in 2019, while coverage in rural areas increased from 61.5% to 82.7% and coverage in tribal lands increased from 57.8% to 79.1% [14].

Efforts to bridge the digital divide often involve government initiatives, private sector investments, and nonprofit organizations working to improve internet infrastructure, provide digital literacy training, and distribute digital devices to underserved populations [15]. Additionally, policies aimed at ensuring affordable internet access and promoting digital inclusion are crucial in addressing this issue [16]. The digital divide is not only a matter of access to technology but also a matter of social equity and inclusion [16].

Reducing the digital divide is essential for ensuring that all individuals and communities have equal opportunities to participate in the digital age and benefit from the numerous opportunities it offers, such as telehealth. Given that a significant portion (approximately 35%) of Nebraska’s population resides in nonmetropolitan areas, this state offers a unique opportunity for in-depth analysis of health care access challenges inherent to rural settings. While 56% of Nebraska’s 1.96 million inhabitants live in 3 metropolitan counties in the eastern part of the state, large portions of the state are sparsely populated [17], and 13 of the 93 counties lacked any actively practicing primary care physician in 2021 [18]. Nebraska has 120 areas designated by the Health Resources and Services Administration as health professional shortage areas for primary care and 88 designated medically underserved areas or populations [19]. Taken together, these statistics suggest that Nebraska might benefit from expanded telehealth to help bridge structural barriers to receipt of general medical care. The aim of this study was to assess the telehealth utilization gap during the COVID-19 pandemic in Nebraska.

## Methods

### Ethical Considerations

The protocol for this study was reviewed by the University of Nebraska Medical Center Institutional Review Board (605-20-EX) and deemed exempt from oversight under 45 CFR 46:104(d), category 2. The cover letter provided information about the study, and completion of the survey indicated voluntary informed consent to participate. Responses were anonymous, and data were scrubbed of associated addresses before the Bureau of Sociological Research (BOSR) provided it to the researchers. A US $1 cash incentive was included in the initial mailing with no further compensation provided.

### Study Design

This was a cross-sectional, mailed survey about health care access.

### Sample Design

To try to ensure broad representation of adult Nebraskans by urbanicity level, a stratified address-based sampling process used the 2013 rural-urban continuum codes to classify counties as urban large, urban small, and rural. To try to increase representation by race and ethnicity, we oversampled census tracts with at least 30% African American (urban large), 30% Hispanic (statewide), or 30% Native American (rural) residents. Assuming an estimated 25% response rate based on prior BOSR studies, 5300 addresses were purchased to sufficiently power the analysis. The detailed methodology report is available in Multimedia Appendix 1.

### Recruitment and Data Collection

The survey methods have been described elsewhere but are briefly summarized here [20]. BOSR was contracted for data collection and purchased Nebraska residential mailing addresses from Dynata. The number and timing of mailings were based on professional recommendations from BOSR. An initial survey packet was mailed to selected addresses in October 2020. The packet (English and Spanish) invited the adult (aged 19 years or older) with the next birthday to complete a web-based survey using a link to Qualtrics. A reminder postcard was sent 2 weeks later, and a full survey packet (English and Spanish) was sent 3 weeks thereafter containing a cover letter, survey, and postage-paid return envelope. Due to lower-than-expected response rates, an additional mailing (English) was sent to nonrespondents in early January 2021 consisting of a reminder letter with a web link, the survey, and a postage-paid return envelope. The survey remained open through early March 2021 for any additional responses. BOSR staff used double entry of paper returns for quality control and sent to the research team a cleaned datafile scrubbed of addresses and including survey weights.

### Measures

The outcome measure was ever use of telehealth (yes or no), worded as “Telehealth is a broad term referring to provision of health education and medical services through telecommunications technology. It includes remote monitoring of vital signs, consultation, evaluation, diagnosis, and prescription. Have you ever used telehealth?”

Sociodemographic variables included age group calculated from year born (<45, 45-64, and ≥65 years); sex (male and female); race (White only, Other, and Not reported) reclassified from “mark all that apply” with “prefer not to answer” added to nonresponse; Hispanic ethnicity (yes or no); marital status (married or partnered versus divorced, separated, widowed, or single); highest education level completed (≤high school graduate, some college, and bachelor’s degree or above); annual household income (<US $50,000, US $50,000-US $74,999, >US $75,000); health insurance (Medicaid, Medicare, Private, Other, and None) recoded from “mark all that apply” to sequentially assign to mutually exclusive categories; and rural or urban status.

A checklist of chronic health conditions (heart condition, high blood pressure, diabetes, lung disease, arthritis, stroke, cancer, depression, and anxiety) was recoded as any versus none.

Health care utilization was assessed for routine checkup, worded as “A routine checkup is a general physical exam, not an exam for a specific injury, illness, or condition. About how long has it been since you last saw a doctor or other health care professional for a routine checkup?” Responses were dichotomized as having a visit within the past 12 months (yes or no). Additional questions asked about seeing a health care provider in the past 12 months for chronic, acute, and mental and behavioral health conditions, dichotomized as (yes or no). Because measures of utilization were highly correlated (*P*<.001), they were then combined into a measure of any health care visit in the past 12 months (yes or no).

Access issues included having a primary care provider, travel time, and internet connectivity. Respondents indicated if they had one or more people they identified as a primary health care provider (yes or no). Estimated average 1-way travel time to medical appointments had 5 response options (<5 min, 5-9 min, 10-19 min, 20-29 min, and 30+ min). Household internet access was assessed overall and based on modality of mobile device and broadband (yes or no for each question).

### Statistical Analysis

Analysis was conducted using SAS (version 9.4; SAS Institute Inc.). Survey weights accounting for the stratified sampling design were used for all analyses. Chi-square tests compared telehealth use based on participant sociodemographic, health, and utilization and access variables. Item nonresponse was addressed with case-wise exclusion, except when addressed by recoding. Measures of utilization were assessed individually and tested for correlation before being combined into a summary variable. We tested for correlation between urbanicity and internet access to avoid potential issues with multicollinearity in modeling. Because chronic conditions are more frequent in older persons, we tested for interaction between age group and chronic conditions. Crude and adjusted robust Poisson regression models computed prevalence odds ratios (POR) with 95% CIs. Variables significant in bivariate analysis were carried into a fully adjusted model. The full model was adjusted for age, sex, ethnicity, education, chronic health condition, any health care visit in the past 12 months, and internet access.

## Results

The response rate was 20.8% (1101/5300). Only 25.5% of adult Nebraskans had ever used telehealth, despite an estimated 97% of the population having internet access (Table 1). In the chi-square analysis, there was not a statistically significant difference in ever use of telehealth between urban (26.4%) and rural (20.8%) residents (*P*=.23). However, internet access and rurality were highly correlated (*P*<.001), with 90.5% of rural respondents reporting internet access compared to 98.3% of urban respondents. Therefore, internet access was used in modeling. Telehealth use was more common among those aged <45 years (32.4%), female individuals (30.7%), non-Hispanic individuals (25.9%), those with at least a bachelor’s degree (32.6%), those who had a routine checkup (30.2%) or health care visit other than a routine checkup (34.2%), and those without any chronic health conditions (29.6%).

**Table 1:** Cross-sectional weighted estimation of the proportion of Nebraska adults who had ever used telehealth by March 2021.

			Unweighted responses (n=1101), n (%)		Total weighted %		Telehealth (%)				Chi-sq *P* value
**Characteristics**							Yes (25.5%)		No (74.5%)		
**Rurality (missing=0)**											.23
	Urban	639 (58.04)		82.72		26.43		73.57			
	Rural	462 (41.96)		17.28		20.84		79.16			
**Age (missing=72)**											.004
	65+	462 (44.90)		18.29		13.53		86.47			
	45-64	348 (33.82)		35.64		22.91		77.09			
	<45	219 (21.28)		46.07		32.41		67.59			
**Sex (missing=51)**											.02
	Male	397 (37.81)		48.63		18.99		81.01			
	Female	653 (62.19)		51.37		30.72		69.28			
**Race (missing=0)**											.57
	White only	893 (81.11)		89.28		26.12		73.88			
	Others	96 (8.72)		4.25		18.92		81.08			
	Not reported	112 (10.17)		6.47		20.68		79.32			
**Ethnicity (missing=63)**											<.001
	Hispanic	54 (5.20)		3.59		3.82		96.18			
	Non-Hispanic	984 (94.80)		96.41		25.93		74.07			
**Marital status (missing=51)**											.07
	Married or couple	591 (56.29)		70.16		27.85		72.15			
	Divorced, separated, widowed or never married	459 (43.71)		29.84		18.65		81.35			
**Highest education completed (missing=51)**											.003
	Bachelor’s or above	366 (34.86)		51.94		32.61		67.39			
	Some college	342 (32.57)		27.74		20.75		79.25			
	<High school	342 (32.57)		20.32		13.84		86.16			
**Annual household income (missing=129)**											.15
	<$50,000	485 (49.90)		28.24		18.66		81.34			
	$50,000-$74,999	177 (18.21)		17.62		26.14		73.86			
	≥$75,000	310 (31.89)		54.15		29.02		70.98			
**Insurance status (missing=33)**											.24
	Medicaid	99 (9.27)		5.69		21.54		78.46			
	Medicare	441 (41.29)		18.73		15.27		84.73			
	Private	461 (43.16)		67.47		29.14		70.86			
	Other	39 (3.65)		5.48		24.67		75.33			
	None	28 (2.62)		2.64		19.30		80.70			
**Chronic health conditions (missing=3)**											.03
	None	266 (24.23)		36.99		18.38		81.62			
	Any	832 (75.77)		63.01		29.62		70.38			
**Routine checkup in past 12 months (missing=21)**											.03
	Yes	703 (65.09)		58.81		30.15		69.85			
	No	377 (34.91)		41.19		18.95		81.05			
**Non-routine health care visit in past 12 months (missing=0)**											.002
	Yes	486 (44.14)		40.58		34.22		65.78			
	No	615 (55.86)		59.42		19.46		80.54			
**Has a primary health care provider (missing=24)**											.13
	Yes	943 (87.56)		82.01		27.53		72.47			
	No	134 (12.44)		17.99		16.55		83.45			
**Average time 1-way for medical travel (missing=23)**											.33
	<5 min	159 (15.17)		11.17		12.72		87.28			
	5-9 min	233 (22.23)		28.47		26.92		73.08			
	10-19 min	318 (30.34)		37.91		28.42		71.58			
	20-29 min	164 (15.65)		13.63		26.37		73.63			
	30+ min	174 (16.60)		8.82		25.68		74.32			
**Any household internet access (missing=47)**											<.001
	Yes	924 (87.67)		96.95		26.34		73.66			
	No	130 (12.33)		3.05		2.95		97.05			
**Internet via mobile device (missing=86)**											.002
	Yes	805 (79.31)		92.87		26.24		73.76			
	No	210 (20.69)		7.13		5.30		94.70			
**Internet via broadband (missing=100)**											.009
	Yes	735 (73.43)		87.57		27.20		72.80			
	No	266 (26.57)		12.43		11.74		88.26			

In univariate models, internet access, younger age groups, female sex, non-Hispanic ethnicity, higher education, any chronic health conditions, and any care in the past year were significantly associated with ever use of telehealth (Table 2). In contrast, race, marital status, some college, income, health insurance, and having a primary care physician were not associated. Travel time was significant but had such a wide confidence interval that it was excluded from further analysis.

**Table 2:** Univariate prevalence odds ratios (POR) and 95% CI of ever use of telehealth, Nebraska adults, cross-sectional survey conducted from October 2020 to March 2021.

Variable		POR	95% CI	*P* value
**Internet**		8.94	3.28-24.38	<.001
**Age group (years)**				
	<45 vs 65+	4.06	1.73-9.54	.03
	45-64 vs 65+	6.88	1.86-25.40	<.001
**Female vs male sex**		1.62	1.06-2.48	.03
**Race**				
	Other vs White	0.57	0.19-1.74	.48
	Not reported vs White	0.42	0.06-2.84	.45
**Non-Hispanic vs Hispanic ethnicity**		6.79	2.50-18.43	<.001
**Married (yes vs no)**		0.67	0.42-1.06	.09
**Education**				
	Some college vs ≤high school	3.53	1.11-11.19	.22
	Bachelor vs ≤high school	5.29	0.90-31.24	.004
**Income (US $)**				
	50,000 to <75,000 vs ≥75,000	0.58	0.27-1.25	.68
	<50,000 vs ≥75,000	0.52	0.15-1.76	.05
**Insurance**				
	Medicaid vs private	0.39	0.12-1.26	.11
	Medicare vs private	0.29	0.03-2.58	.26
	Other vs private	0.12	0.001-24.16	.43
	None vs private	0.39	0.12-1.26	.11
**Chronic condition (yes vs no)**		1.61	1.03-2.52	.04
**Primary care physician (no vs yes)**		0.60	0.30-1.22	.16
**Any health care visit (routine or otherwise) in past 12 months (yes vs no)**		2.64	1.39-5.01	.003
**Average time to nonemergency health care (min)**				
	5-9 vs <5	10.02	1.29-77.66	.04
	10-19 vs <5	21.22	1.34-336.37	.02
	20-29 vs <5	44.92	1.38-1464.43	.07
	30+ vs <5	95.10	1.42-6391.80	.08

**Figure 1:**
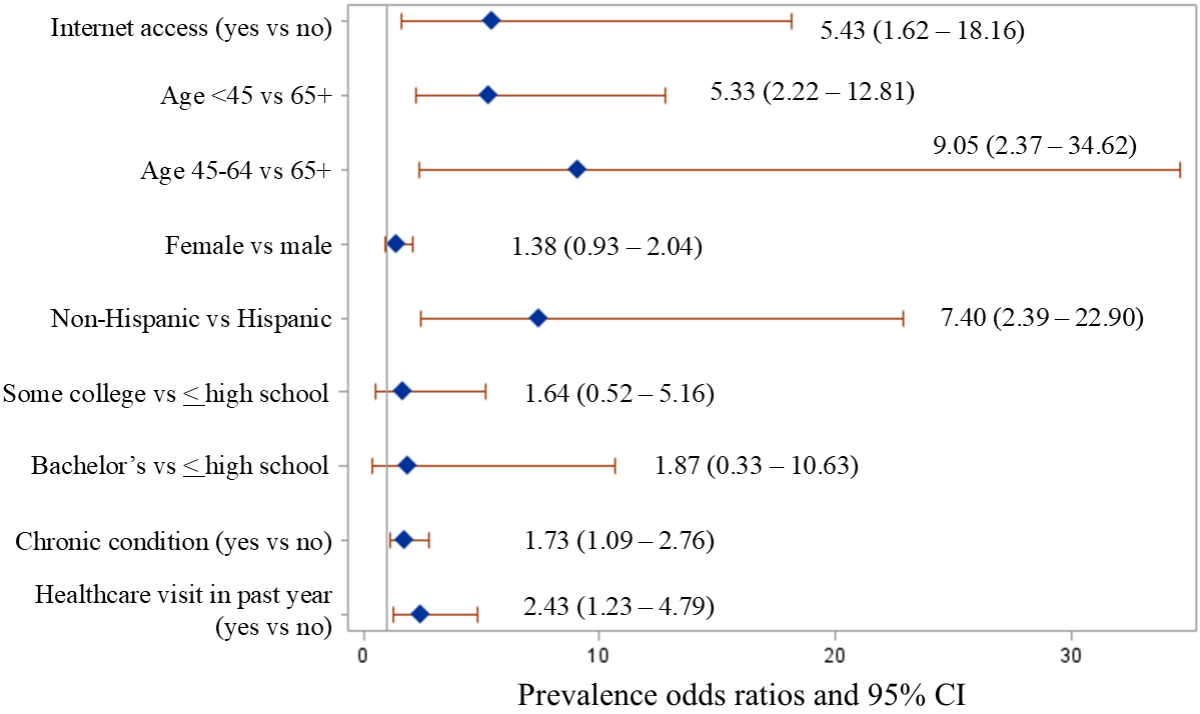
Prevalence odds ratios (95% CI) of the final survey-weighted adjusted model of ever use of telehealth in Nebraska, October 2020 to March 2021.

In multivariate modeling, chronic condition by age interaction was not significant. In the final adjusted model, education (POR 1.87, 95% CI 0.33-10.63) and sex (POR 1.38, 95% CI 0.93-2.04) were not significant, but internet access (POR 5.43, 95% CI 1.62-18.16), age <45 (POR 5.33, 95% CI 2.22-12.81) and 45-64 years (POR 9.05, 95% CI 2.37-34.62), non-Hispanic ethnicity (POR 7.40, 95% CI 2.39-22.90), any chronic condition (POR 1.73, 95% CI 1.09-2.76), and any health care appointment in the past 12 months (POR 2.43, 95% CI 1.23-4.79) were significantly associated with having ever used telehealth (Figure 1).

## Discussion

### Principal Findings

During the first year of the COVID-19 pandemic, this study focused on evaluating the utilization of telehealth in Nebraska, a primarily rural state. Although most respondents had internet access, we found that the availability and accessibility of the internet played a significant role in the utilization of telehealth services. While the estimate of adults in Nebraska who had ever used telehealth is relatively low, having internet access was a significant predictor of using those services. The study further highlighted a strong correlation between internet access and rurality, suggesting that individuals in rural areas may encounter greater barriers when trying to access telehealth services. These findings highlight the digital divide as a barrier to telehealth, with internet access being a strong predictor of telehealth usage.

In addition, disparities in telehealth usage were identified based on age, chronic health conditions, ethnicity, and health care utilization. Older adults, individuals without chronic health conditions, and people of Hispanic ethnicity were significantly less likely to have ever used telehealth compared to other demographic groups. These disparities highlight the need for targeted support to ensure equitable access and utilization of telehealth services, potentially informing strategies to address barriers related to internet access and demographic factors. Unsurprisingly, having a health care visit in the past year was associated with use of telehealth.

Our study supports prior research that found geographical location plays a part in inequities between different populations. In our study, the overall population had relatively high rates of any type of internet access (broadband or mobile network), but urban areas had better access than rural areas; our rural population reported better access than the FCC report [14], although our unweighted results were similar to the FCC report for rural areas but lower for urban areas. Similarly, another study found a positive association between increased broadband access and telemedicine use, but only in counties classified as fully rural [21]. High expense and low profit margins deter investment in expanding internet access to rural communities [22-24] and minority households [25]. Furthermore, the anticipated higher costs of internet in rural areas made it unlikely that lower economic residents could afford the high cost of the internet [26].

Consistent with some previous research, our study reinforces evidence that telehealth utilization for internet-based health care services is more prevalent among young adults [7,27,28], non-Hispanic White individuals [7,28-30], those with higher levels of education, and female individuals [7,27]. These demographic characteristics have consistently emerged as influential factors in determining telehealth usage across multiple studies. In contrast to these findings, Hong and Cho [12] found that in 2011, those aged 75+ years were more likely to have communicated with health care professionals online, and education became less significant from 2003 to 2011, indicating a narrowing of the digital divide in regard to age and education. The differences in the data are likely the result of the broad definition of communication used by Hong and Cho [12] versus our definition of telehealth as health education and medical services through telecommunications, with an emphasis on medical services.

Our research presents a contrasting perspective when compared to previous studies in the field. Other investigations have found linkage between higher socioeconomic status [7] and Medicare/Medicaid insurance status [28] with increased telehealth utilization. These may be attributed to various factors, including greater access to the necessary technology, higher levels of health literacy, and more comprehensive insurance coverage, which includes coverage for telehealth services. However, in our analysis, these associations did not emerge as significant. This discrepancy may imply that barriers to telehealth access are addressed in ways that reduce disparities in relation to these factors. Additional research is warranted to support the demographic shift toward more equitable telehealth utilization and to explore the role that COVID-19 has played in the adoption of telehealth services.

Furthermore, our findings highlight a noteworthy association between the presence of chronic health conditions and a higher likelihood of using telehealth services. Older adults are more likely to experience chronic health conditions and less likely to access the technology necessary to use telehealth video [29], although age group interaction with chronic conditions was not significant in our study. This suggests that individuals with chronic conditions are more likely to engage in telehealth consultations compared to those without such conditions. However, Cousins et al [28] noted increased telehealth usage among older adults during the COVID-19 pandemic, while another study noted an increase in comfort among older adults with telehealth usage [31]. It is important to recognize that telehealth has the potential to support improved outcomes for individuals with chronic conditions, as it has been found to be a safe alternative to traditional care [32], enhancing patient education [33], facilitating disease management [34], and supporting self-management [32,34]. While it may not completely replace in-person visits, telehealth can supplement traditional care by facilitating remote monitoring, providing timely access to health care professionals, and enabling convenient follow-up consultations [6].

Disparities in access to telehealth services exist among older adults, marginalized racial or ethnic communities, and rural residents. Familiarity with communication devices and internet usage can also impose limitations on telehealth access, particularly for older adults. In addition, replacing a percentage of traditional health care services with telehealth access may exacerbate existing health disparities by favoring younger, employed, and urban-dwelling patients [35].

In the broader context of our findings, the emphasis on Nebraska underscores the need for tailored interventions to address telehealth disparities. The state’s considerable rural demographic provides a valuable perspective on the unique challenges and opportunities telehealth offers in rural America. Rural areas face unique challenges, including access to health care, education, and economic opportunities, which are indicative of the declining populations in many rural counties. By focusing on Nebraska, we illustrate the pressing need for enhancements in telehealth infrastructure, policy adjustments, and community centered initiatives to eliminate barriers and improve telehealth access. Our investigation into Nebraska’s telehealth landscape offers valuable insights into the challenges and resilience of rural communities, not just in Nebraska but across the Great Plains and the broader United States.

### Limitations

In this study, sampling weights accounted for the oversampling of certain census tracts and were used to make the responses representative of the Nebraska population by sex and age. However, Nebraska lacks racial diversity (non-Hispanic White residents represent 77% of the population), and racial or ethnic groups are not evenly distributed across the state. Although we attempted to obtain diverse responses, we were unable to apply sampling weights for race. Weighting actually diluted the responses for certain groups. Therefore, we lacked the diversity that we intended and were unable to make more in-depth comparisons beyond White (yes vs no). We specifically listed the reference group as “White only” because respondents were allowed to select multiple racial categories to self-identify. The absence of race-based sampling weights means that the sample may not accurately reflect the racial composition of the population, limiting the study’s external validity. Furthermore, the weighting process employed in the study appeared to dilute responses from rural areas due to oversampling. Approximately 35% of Nebraska’s population is rural, but 42% of our respondents were rural and 17% were from small urban locations. Given that rural areas often possess unique characteristics, such as different socioeconomic conditions, limited access to resources, and specific cultural factors, the dilution of responses from these regions can compromise the study’s ability to capture the full range of perspectives and experiences. Future research should aim to address these limitations by employing more comprehensive sampling strategies that account for a wider range of demographic variables and ensure adequate representation across various geographic locations. Another limitation of the study revolved around the issue of item nonresponse, which further diluted our multivariate models. This issue may weaken the reliability of our multivariate models. The number of health care visits for chronic, acute, and mental and behavioral health conditions was recorded as a write-in response, which resulted in potential misclassification; several respondents reported more than 30 visits in the past 12 months. Therefore, we dichotomized as any versus none for analysis. Future studies may want to consider the types of visits and frequency of visits for their impact on exposure to telehealth as a service delivery method.

### Conclusions

Our investigation into telehealth utilization during the onset of the COVID-19 pandemic shows the need for targeted strategies to bridge the digital divide and enhance telehealth access, especially in rural areas. At a time when the pandemic dramatically increased the need for telehealth services, serving as an essential tool for patients unable to visit medical professionals in person, our study reveals that disparities in telehealth usage were already present. The challenges identified in Nebraska’s communities highlight how demographic factors, such as age, chronic health conditions, ethnicity, and especially internet accessibility, affect telehealth utilization, highlighting the preexisting barriers to equitable health care delivery. These findings further emphasize the continued need for policy and infrastructure reform to address these disparities. Moving forward, it is important to use these insights to guide the development of inclusive telehealth programs and policies that are sensitive to the diverse needs of all populations, ensuring that telehealth can fulfill its potential as a cornerstone of accessible and equitable health care.

## Appendix

Multimedia Appendix 1 Methodology report from the Bureau of Sociological Research (BOSR).

## Abbreviations

BOSR: Bureau of Sociological Research
FCC: Federal Communications Commission
POR: prevalence odds ratio

## Data Availability

The dataset used and analyzed during the current study is available from the corresponding author on reasonable request.
