# Supplementary material for: Ever Use of Telehealth in Nebraska by March 2021: Cross-Sectional Analysis
Source: J Med Internet Res. 2024 Nov 28;26:e53320. doi: 10.2196/53320 (PMC11638684; doi:10.2196/53320)
Supplement: Multimedia Appendix 1 [file jmir_v26i1e53320_app1.pdf]

BOSR

Bureau of Sociological Research

Health and Health Care Access in Nebraska

Prepared: March 2021

ASSOCIATION OF ACADEMIC SURVEY RESEARCH ORGANIZATIONS AASRO

Charter Member AAPOR Transparency Initiative AMERICAN ASSOCIATION FOR PUBLIC OPINION RESEARCH

The contents of this report conform to our highest standards for data collection and reporting. If you should have any questions or concerns regarding the information reported within, please contact us.

Bureau of Sociological Research
Department of Sociology
PO Box 880325 • Lincoln, NE 68588-0325
402-472- 3672 (local) • 800-480- 4549 (toll free)
email: bosr@unl.edu • http://bosr.unl.edu

Contents

Introduction 4

Sampling Design 4

Questionnaire Design 4

Data Collection Process 4

Response Rate 5

Data Processing 5

Data Cleaning 6

Data Weights 6

Design Effect 7

Questions 7

Appendices 8

- Appendix A: Survey Instrument 8
  - Paper - English 8
  - Paper - Spanish 16
  - Web - English 24
  - Web - Spanish 56
- Appendix B: Communications 91
  - Invitation - English 91
  - Invitation - Spanish 92
  - Reminder - English 93
  - Reminder - Spanish 94
  - Final Reminder - English 95
  - Final Reminder - Spanish 96
  - Additional Mailing 97
- Appendix C: Strata 98
  - Stratum 1: Urban large counties without oversample 98
  - Stratum 2: Urban small counties 98
  - Stratum 3: Rural counties 98
  - Stratum 4: Oversample census tracts where at least 30% of the population is African American 98
  - Stratum 5: Oversample census tracts where at least 30% of the population is Native American 99
  - Stratum 6: Oversample census tracts where at least 30% of the population is Hispanic 99
- Appendix D: Estimate of Sampling Error 100

Appendix E: AAPOR Transparency Initiative Immediate Disclosure Items 101

Introduction

The purpose of this study is to understand the health and history of health conditions for Nebraskans all over the state. The survey was also designed to help the researcher figure out the insurance coverage of Nebraskans and any issues they had obtaining coverage. This project was sponsored by a faculty member from the College of Nebraska Medical Center (UNMC) and the survey was conducted by the Bureau of Sociological Research (BOSR). Users of the survey data will find this an important reference source for answers to questions about methodology.

Sampling Design

Dynata provided addresses of 5,300 households selected through address-based sampling (ABS) in Nebraska. There were three levels of urbanicity and three oversamples. Two of the oversamples were within two of the urbanicity levels with the final oversample being statewide. Two oversamples targeted census tracts that had at least 30% African American or Native American residents. Because none of the census tracts in the urban small region had census tracts with more than 30% of these populations, an oversample of these populations was not taken for this region. The final oversample had census tracts with more that 30% Hispanic residents and came from tracts in all urbanicities. This resulted in six strata. The first level of urbanicity had 841 residents with the other two containing 842 residents. The three oversamples had 925 residents. A full list of the strata can be found in Appendix C. The sample was cleaned by BOSR project staff.

Questionnaire Design

The final questions were determined as the outcome of a joint effort between researchers of this project at UNMC and BOSR. After rounds of edits, the questionnaire that was produced had two versions, a Spanish one and an English one. Both of these were eight-pages. The survey had a total of 92 questions, which addressed multiple substantive topics focused on health care and Nebraskans' access to it, as well as one section asking questions on respondent's demographics specifically. The English version was translated into Spanish using the Modern Languages and Literatures Department at the University of Nebraska – Lincoln (UNL). A web component of the survey was created using Qualtrics which was also in English and Spanish. A copy of the survey can be found in Appendix A.

Data Collection Process

The data collection process involved four mailings. In the initial contact, a cover letter with English on one side and Spanish on the other, was sent to respondents explaining the survey that also included the link to the Qualtrics survey. The initial invitation also included a $1 incentive and was sent on October 27, 2020. For each address, the adult age 19 or older of that household who would have the next birthday after October 1, 2020 was asked to complete the questionnaire. One week after the first mailing, all households were mailed a postcard reminding them to complete the survey or expressing appreciation if they had already completed the survey. This reminder postcard was sent on November 10, 2020. Finally nonrespondents received a survey package, which contained the cover letter, two paper copies of the survey one in English and one in Spanish, along with a postage pre-paid addressed business reply envelope for the survey to be mailed back to BOSR. The final mail survey package was

sent out on December 1, 2020. Due to a low response rate by respondents an additional mailing was sent out. This mailing consisted of a survey package, which contained the cover letter, a single copy of the survey, along with a postage pre-paid addressed business reply envelope for the survey to be mailed back to BOSR. The additional mailing was only in English. All communication materials were in English and Spanish and can be found in Appendix B.

Response Rate

In total, 1,101 surveys were completed or partially completed by the end of the field period on March 8, 2021. The response rate of 20.8% was calculated using the American Association for Public Opinion Research's (AAPOR) standard definition for Response Rate 2. Of the 5,300 addresses sampled, 8.1% (n=430) were determined to be ineligible (e.g., no such address; vacant) and 6.4% (n=341) were undeliverable addresses with unknown eligibility. Refusals (e.g., blank survey returned; letter, phone call, or e-mail stating refusal to participate) and refused mail were obtained from 0.7% (n=36) of the sample. Table 1 shows the response rates by stratum.

Table 1. Response rates by stratum

Stratum	AAPOR Response Rate 2	n
Urban large	3.8%	201
Urban small	3.9%	207
Rural	3.9%	207
African American oversample	2.4%	128
Native American oversample	4.4%	231
Hispanic Oversample	2.4%	127

Data Processing

Mail survey data were entered using Epi Info 6 software with data saved on BOSR's secure networked file server. Data entry was completed by experienced data-entry staff. All of the data-entry workers had previous experience in data entry using Epi Info 6 on other mail survey projects. The data-entry staff was supervised by full-time BOSR project staff.

Mail data entry was completed in two steps. First, one data-entry worker would enter responses from a single survey. Second, another data-entry worker would re-key the survey and be alerted to any discrepancies with the first entry. Supervisory staff members were available to answer questions about discrepancies or illegible responses. The data-entry staff is paid by the hour, not by the number of surveys entered. This method of payment is used so that we can ensure the high quality of the data collected by our staff.

Regarding the web data portion of this study, since respondents entered their responses directly into a computerized instrument, this survey required no additional data entry or data processing steps.

The web survey data were recorded in Qualtrics and stored on a secure server located within the Sociology Department at UNL after being exported. The Statistical Package for the Social Sciences (SPSS) software package was used to process and document the dataset. The dataset was exported from Qualtrics into an SPSS system file.

Data Cleaning

The data was recorded and stored on a secure server located within the Sociology Department at UNL. The Statistical Package for the Social Sciences (SPSS) software package was used to process and document the dataset. The dataset was exported from Epi Info 6 into an SPSS system file. BOSR removed any cases that were duplicate or blank. The first step in data cleaning was to run frequency distributions on each of the variables in the survey. The second step was to generate variable and value labels. The final step in data cleaning was to check for out-of-range values on all survey items. Recoding was done to correct for the most obvious errors/inconsistencies in the data.

The first step in data cleaning for the web data was to delete any variables that had identifying information. The second step was to check variable and value labels. Both datasets were merged into one.

Data Weights

In order to make the data statistically representative of the state-wide population, weights were created for the data. The data were weighted in four ways to account for the address probability of selection, nonresponse, within household probability of selection, and population characteristics. First, data were weighted by stratum in order to account for the disproportionate stratified sample design (sampwat). As stated above, six separate samples with different probabilities of selection were drawn. Then, the data were weighted by the number of adults living in the household (Hwat) in order to adjust for within-household selection probability. Then, the data were weighted for nonresponse (NRweight) to account for the difference in response rates by stratum. Lastly, poststratification weights were applied based on age (age_grp) and gender (sex) in order for the data to more closely resemble the population (post_cat). Table 2 shows the poststratification groups and the population counts from the 2018 American Community Survey (ACS) for each group. The population data age category for early adults includes 18 year olds. However, the age of majority in Nebraska is 19 years old meaning that the given age categories do not perfectly provide the necessary data. As a result, the number of 19 year olds was calculated as 1% of the overall Nebraska population.

Table 2. Population counts by age group and sex

	19-44	45-64	65+	Total
Male	310,945	233,200	105,687	649,832
Female	303,292	236,189	139,340	678,821
Total	614,237	469,389	245,027	1,328,653

Table 3 displays population frequencies and its comparison to this survey data's weighted and unweighted frequencies. Sampling (sampwat), nonresponse (NRwt), and poststratification (post_cat) weights were multiplied together and rescaled (Rescale) to create the final weight. The final weight in the dataset is called Pwate.

Table 3. Representativeness of 2021 Health and Health Care Access in Nebraska Survey Sample by Age and Sex (Percentage Distribution in Age and Sex Categories)*

Category	Based on 2010 Census Estimate	Social Health, Unweighted	Social Health, Weighted By Pwate
Age Group:			
19 – 64	81.6%	55.2%	81.8%
65+	18.4%	44.8%	18.2%
Sex:			
Males	48.9%	37.8%	48.6%
Females	51.1%	62.2%	51.4%
Total	100%	100%	100%

*Weighted estimates are calculated using imputed variables. The frequencies above are of the variables before imputation. As a result, the weighted frequencies do not exactly match the 2010 Census estimates.

Design Effect

The design effect due to overall weighting adjustments is 3.40, which represents the loss in statistical efficiency that results from unequal weights[1].

Disproportionate stratification was used for the Health and Health Care Access in Nebraska Survey, as discussed earlier. The use of this type of sampling resulted in a sampling design effect of 0.34[2].

Appropriate adjustments need to be incorporated into statistical tests when using the Health and Health Care Access in Nebraska Survey data. See Appendix D for more information.

Questions

Any questions regarding this report or the data collected can be directed to the Bureau of Sociological Research at the University of Nebraska-Lincoln by calling (402) 472-3672 or by sending an e-mail to bosr@unl.edu.

[1] The formula used is: $1 + cv^2(w) = \frac{n(\sum_1^n w_i^2)}{(\sum_1^n w_i)^2}$

[2] The formula used is: $deff = \frac{\text{var}_{complex}(\bar{y})}{\text{var}_{SRS}(\bar{y})}$. Used Q1 (Would you say that in general your health is excellent, very good, good, fair, or poor?) to calculate.

Appendices

Appendix A: Survey Instrument

Paper - English

Health and Health Care Access in Nebraska

Health Status

1. Would you say that in general your health is...
   - ◯ Excellent
   - ◯ Very good
   - ◯ Good
   - ◯ Fair
   - ◯ Poor

2. Thinking about your physical health, which includes physical illness and injury, for how many days during the past 30 days was your physical health NOT good?

   ☐☐ days

3. Thinking about your mental health, which includes stress, depression, and problems with emotions, for how many days during the past 30 days was your physical health NOT good?

   ☐☐ days

4. During the past 30 days, for how many days did poor physical or mental health keep you from doing your usual activities, such as self-care, work, or recreation?

   ☐☐ days

5. Has a doctor or other health professional ever told you that you had any of the following medical conditions?

	Yes	No
a. COVID-19 (coronavirus)	◯	◯
b. Heart condition (heart attack, heart disease, myocardial infraction, angina, congestive heart failure)	◯	◯
c. High blood pressure (hypertension)	◯	◯
d. Diabetes, pre-diabetes, borderline diabetes, or high blood sugar	◯	◯
e. Lung disease (COPD, chronic lung disease, emphysema, chronic bronchitis, or asthma)	◯	◯
f. Arthritis (including osteo or rheumatoid arthritis) or rheumatism	◯	◯
g. Stroke	◯	◯
h. Cancer (please specify) ☐	◯	◯
i. Depression or anxiety disorder	◯	◯

6. In the past 12 months, have you had an overnight hospital stay?
   - ◯ Yes
   - ◯ No

7. Have you ever had any of the following types of surgery?

	Yes	No
a. Knee repair or replacement surgery	◯	◯
b. Hip repair or replacement surgery	◯	◯
c. Back or spine surgery	◯	◯
d. Heart surgery of any kind, including a bypass, valve surgery, or stent	◯	◯

8. How much difficulty do you have walking or climbing steps?
   - ◯ No difficulty
   - ◯ Some difficulty
   - ◯ A lot of difficulty
   - ◯ Cannot do at all

Health Behaviors

9. During the past month, other than your regular job, did you participate in any physical activities or exercises? *Examples might include running, calisthenics, golf, gardening, or walking for exercise.*
   - ◯ Yes
   - ◯ No

10. During the past 12 months, have you had either a flu shot or a flu vaccine that was sprayed in your nose?
    - ◯ Yes
    - ◯ No

11. Have you smoked at least 100 cigarettes (5 packs) in your lifetime?
    - ◯ Yes
    - ◯ No

12. Have you ever used an electronic cigarette or other elecrontic vaping products (such as e-cigarette, vape, Juul, NJOY, Blu), even just once in your entire life?
    - ◯ Yes
    - ◯ No

13. During the past 30 days, how many days did you have at least one drink of any alcoholic beverage such as beer, wine, a malt beverage or liquor?

    ☐☐ days

The next questions are about cancer screening…

14. A blood stool test is a test that may use a special kit at home to determine whether the stool contains blood. Have you ever had this test?
    - ○ Yes
    - ○ No→ Go to #16

15. When was your most recent blood stool test?

    Month ☐☐ Year ☐☐☐☐

16. Sigmoidoscopy and colonoscopy are exams in which a tube is inserted in the rectum to view the colon for signs of cancer or other health problems. Have you ever had either of these exams?
    - ○ Yes
    - ○ No→ Go to #18

17. When was your most recent sigmoidoscopy or colonoscopy?

    Month ☐☐ Year ☐☐☐☐

18. Have you ever been screened for lung cancer?
    - ○ Yes
    - ○ No→ Go to #20

19. When was your most recent lung cancer screening?

    Month ☐☐ Year ☐☐☐☐

20. For women: Have you ever had a mammogram?
    - ○ Yes
    - ○ No→ Go to #22

21. When was your most recent mammogram?

    Month ☐☐ Year ☐☐☐☐

22. For women: Have you ever had a Pap test?
    - ○ Yes
    - ○ No→ Go to #24

23. When was your most recent Pap test?

    Month ☐☐ Year ☐☐☐☐

24. For men: Have you ever been screened for prostate cancer (such as the Prostate-Specific Antigen or PSA test)?
    - ○ Yes
    - ○ No→ Go to #26

25. When was your most recent screening?

    Month ☐☐ Year ☐☐☐☐

Health Care Access and Utilization

26. Are you currently covered by any of the following types of health insurance or health coverage plans?

	Yes	No
a. Insurance through a current or former employer or union	○	○
b. Insurance purchased directly from an insurance company (marketplace)	○	○
c. Medicare (for people 65 and older, or people with certain disabilities)	○	○
d. Medicaid, Medical Assistance, or any kind of government-assisted plan for those with low income or a disability	○	○
e. TRICARE (CHAMPUS) or other military health care	○	○
f. VA (including those who have ever used or enrolled in VA health care)	○	○
g. Indian Health Service	○	○
h. Any other type of health insurance or health coverage plan (please specify) ________	○	○
i. Do not have health insurance	○	○

27. If you do not currently have health care coverage, about when did you last have coverage?

    Month ☐☐ Year ☐☐☐☐

28. Insurance navigators educate the public about health care plans, tax credits, and cost sharing. They also help enroll people into health benefit plans on the health insurance marketplace. Have you ever used an insurance navigator?
    - ○ Yes
    - ○ No

29. A patient navigator or patient advocate helps guide a patient through the healthcare system and communicate with healthcare providers. A patient navigator also works to overcome obstacles that are in the way of the patient receiving care. Have you ever used a patient navigator or patient care coordinator?
- ◯ Yes
- ◯ No

30. Telehealth is a board term referring to provision of health education and medical services through telecommunications technology. It includes remote monitoring of vital signs, consultation, evaluation, diagnosis, and prescription. Have you ever used telehealth?
- ◯ Yes
- ◯ No

31. Do you have one person you think of as your personal doctor or health care provider?
- ◯ Yes, only one
- ◯ Yes, more than one
- ◯ No, there is no person I think of as my personal doctor or health care provider

32. Do you currently have any health care bills that are being paid off over time?
- ◯ Yes
- ◯ No

33. Was there a time in the past 12 months when you needed to see a doctor but could not because of cost?
- ◯ Yes
- ◯ No

34. Other than cost, in the past 12 months have you delayed getting medical care for any of the following reasons?

	Yes	No
a. You couldn't get through on the phone (busy signal, you were put on a long hold, no one answered)	◯	◯
b. You couldn't get an appointment soon enough	◯	◯
c. Once you got there, you had to wait too long to see the doctor	◯	◯
d. The clinic or doctor's office wasn't open when you got there	◯	◯
e. You didn't have transportation	◯	◯
f. You didn't have care for another household member (childcare, eldercare)	◯	◯
g. You couldn't take time off from work	◯	◯
h. Language barriers	◯	◯

35. Other than the past 12 months, did you ever put off receiving health care because you were concerned about cost?
- ◯ Yes
- ◯ No

36. Other than the past 12 months, did you ever put off receiving health care because of distance or transportation concerns?
- ◯ Yes
- ◯ No

37. Not including over the counter medications, was there a time in the past 12 months when you did not take your medication as prescribed because of cost?
- ◯ Yes
- ◯ No
- ◯ Not applicable (no medication was prescribed)

38. Have you ever had difficulty filling a prescription because you had no reliable transportation?
- ◯ Yes
- ◯ No
- ◯ Not applicable (no medication was prescribed)

39. A routine checkup is a general physical exam, not an exam for a specific injury, illness, or condition. About how long has it been since you last saw a doctor or other health care professional for a rountine checkup?
- ◯ Less than 12 months ago
- ◯ 1 year to less than 2 years ago
- ◯ 2 years to less than 3 years ago
- ◯ 3 years to less than 5 years ago
- ◯ 5 years or more
- ◯ Never

40. In the past 12 months, how many times have you seen a health professional for…

a. Chronic condition (such as arthritis, diabetes, heart disease, cancer, or asthma)?	☐	Number of visits
b. Acute condition (such as heart attack, broken bone, injury, sudden fever, severe chest pains, severe asthma attack)?	☐	Number of visits
c. Mental or behavioral health?	☐	Number of visits

41. In the past 12 months, how many times have you been to a cancer-related appointment (screening, diagnosis, treatment, or follow-up)?

[ ] Number of visits

42. In the past 12 months, how many times have you been to the emergency room?

[ ] Number of visits

43. In the past 12 months, how many times have you accompanied someone else to a medical appointment or visit to a health professional?

[ ] Number of visits

44. In the past 12 months, how many times have you accompanied someone else to a cancer-related appointment (screening, diagnosis, treatment, or follow-up)?

[ ] Number of visits

45. Including all types of dentists (dental hygienist, dentist, orthodontist, oral surgeon, and all other dental specialists), how long has it been since you last visited a dentist or a dental clinic for any reason?

- ○ Less than 12 months ago
- ○ 1 year to less than 2 years ago
- ○ 2 years to less than 3 years ago
- ○ 3 years to less than 5 years ago
- ○ 5 years ago or more
- ○ Never

Problems List

46. Please indicate if any of the following has been a problem for you in the past 30 days.

	Yes	No
a. Child care	○	○
b. Dependent adult care	○	○
c. Unable to work due to disability	○	○
d. Child with unmet special education needs	○	○
e. Reduced work hours (including furlough or lay-off)	○	○
f. Health insurance	○	○
g. Unemployment benefits	○	○
h. Disability benefits	○	○
i. SNAP/WIC	○	○
j. Ran out of food before you could buy more	○	○
k. Couldn't afford to eat balanced meals	○	○
l. Bugs or rodents in household	○	○
m. Lead paint in household	○	○
n. Mold or dampness in household	○	○
o. Threat of eviction	○	○
p. Unreliable utilities	○	○
q. General cleanliness of household	○	○
r. Overcrowding in household	○	○
s. Transportation	○	○
t. Health care (for self or household member)	○	○
u. Legal status (concerns with family immigration status)	○	○
v. Personal and family stability (for example, domestic violence, guardianship or custody issues, child welfare)	○	○
w. Other (please specify) [ ]	○	○

47. Please indicate if any of the following has been a problem for you during 2019.

	Yes	No
a. Child care	◯	◯
b. Dependent adult care	◯	◯
c. Unable to work due to disability	◯	◯
d. Child with unmet special education needs	◯	◯
e. Reduced work hours (including furlough or lay-off)	◯	◯
f. Health insurance	◯	◯
g. Unemployment benefits	◯	◯
h. Disability benefits	◯	◯
i. SNAP/WIC	◯	◯
j. Ran out of food before you could buy more	◯	◯
k. Couldn't afford to eat balanced meals	◯	◯
l. Bugs or rodents in household	◯	◯
m. Lead paint in household	◯	◯
n. Mold or dampness in household	◯	◯
o. Threat of eviction	◯	◯
p. Unreliable utilities	◯	◯
q. General cleanliness of household	◯	◯
r. Overcrowding in household	◯	◯
s. Transportation	◯	◯
t. Health care (for self or household member)	◯	◯
u. Legal status (concerns with family immigration status)	◯	◯
v. Personal and family stability (for example, domestic violence, guardianship or custody issues, child welfare)	◯	◯
w. Other (please specify) ______	◯	◯

Phone and Computer Access

48. Do you or any member of your household own or use any of the following types of computers?

	Yes	No
a. Desktop, laptop, tablet or other portable wireless computer	◯	◯
b. Smartphone	◯	◯

49. Do you or any member of your household have access to the internet?
- ◯ Yes
- ◯ No→ Go to #51

50. Do you or any member of your household have access to the interent using each of the following...?

	Yes	No
a. Cellular data plan for a smartphone or other mobile device	◯	◯
b. Broadband (high speed) internet service such as cable, fiber optic, or DSL service installed at your house	◯	◯
c. Satellite internet service installed at your house	◯	◯
d. Dial-up internet serviced installed at your house	◯	◯

51. How is the cell phone reception at your home?
- ◯ Good
- ◯ Fair
- ◯ Poor

Vehicle Ownership

52. How many vehicles do you or your household members own? Include cars, trucks, SUVs, or vans. *Do NOT include recreational vehicles such as motorcycles, trailers, motor homes, ATV, snowmobiles, boats, or airplanes.*

[|] vehicles

53. How many vehicles are currently working and available for household members to use?

[|] vehicles

54. Do you or your household members own any motorcycles?
- ◯ Yes
- ◯ No → Go to #57

55. How many motorcycles are currently working and available for household members to use?

[|] vehicles

56. You primarily use your motorcycle for...?
- ◯ Recreation
- ◯ Commuting to work or school
- ◯ Running errands

Transportation

57. Do you have a valid driver's license?
- ◯ Yes
- ◯ No

58. Does anyone in your household have a valid driver's license?
- ◯ Yes
- ◯ No

59. Have you ever quit a job or lost a job because you had no reliable transportation to get to work?
- ◯ Yes
- ◯ No

60. Does a lack of transportation options prevent you now from working?
- ◯ Yes
- ◯ No

61. What is the maximum amount you would be willing to pay for a <u>one-way</u> ride to or from work?
- ◯ Less than $1.00
- ◯ $1.00 - $1.99
- ◯ $2.00 - $2.99
- ◯ $3.00 or more

62. What is the maximum amount you would be willing to pay for a <u>one-way</u> ride to or from a medical or health service appointment?
- ◯ Less than $1.00
- ◯ $1.00 - $1.99
- ◯ $2.00 - $2.99
- ◯ $3.00 or more

63. If you have to get somewhere in a car, how difficult is it for you to get there?
- ◯ Very difficult
- ◯ Somewhat difficult
- ◯ Not at all difficult

64. Non-emergency medical transportation (NEMT) is a service for Medicaid beneficiaries who qualify due to transportation needs. It provides transportation to non-emergency but medically necessary appointments. Have you ever used NEMT (for yourself or accompanying someone else)?
- ◯ Yes
- ◯ No→ Go to #66

65. Did you experience any of the following when using NEMT…

	Yes	No
a. Ride transported other passengers at the same time	◯	◯
b. Difficulty scheduling a ride (making reservations)	◯	◯
c. Ride was late	◯	◯
d. Ride did not show up	◯	◯
e. Ride could not accommodate your physical needs (such as wheelchair accessible)	◯	◯
f. Ride took too long because it had to make other stops	◯	◯

66. Do you usually use each of the following to travel to your medical or health care appointments?

	Yes	No
a. Drive yourself	◯	◯
b. Get a ride from a family member or friend	◯	◯
c. Walk (or wheelchair)	◯	◯
d. Bicycle	◯	◯
e. Take public transportation (bus, train, subway)	◯	◯
f. Take a taxi	◯	◯
g. Use a paid ride share service such as Uber or Lyft	◯	◯
h. Use a van or shuttle service provided by the place you live	◯	◯
i. Use a van or shuttle service for seniors or people with disabilities	◯	◯
j. Use transportation provided by the medical facility (such as a car or van, Non-Emergency Medical Transportation)	◯	◯
k. Other (please specify) ______	◯	◯

67. Do you have a relative or friend who regularly provides transportation to a member of your household?
- ◯ Yes
- ◯ No

68. In the past year, did a transportation problem ever keep you from doing the following…

	Yes	No
a. Visiting in person with friends or family not living with you	◯	◯
b. Attending religious services	◯	◯
c. Participating in clubs, classes, or other organized activities	◯	◯
d. Going out for enjoyment (such as going out to dinner, to gamble, to hear music, or to see a movie, play or sporting event)	◯	◯
e. Seeking medical care	◯	◯

69. In the past 30 days, how often did you use the following modes of transportation to get to destinations outside your home?

	7 days/ week	5-6 days/ week	2-4 days/ week	Once a week or less	Never
a. Drive yourself in a personal vehicle (car, SUV, van, pickup truck, motorcycle)	○	○	○	○	○
b. Walk (or wheelchair)	○	○	○	○	○
c. Bicycle	○	○	○	○	○
d. Get a ride from a family member or friend	○	○	○	○	○
e. Take public transportation (bus, train, subway)	○	○	○	○	○
f. Take a taxi	○	○	○	○	○
g. Use a paid ride share service such as Uber or Lyft	○	○	○	○	○
h. Use a van or shuttle service provided by the place you live	○	○	○	○	○
i. Use a van or shuttle service for seniors or people with disabilities	○	○	○	○	○
j. Drive a golf cart, Segway, or riding lawn mower	○	○	○	○	○
k. Drive farm equipment	○	○	○	○	○
l. Ride a horse, donkey or burro or drive a horse-drawn carriage	○	○	○	○	○

Travel Distance

70. Are you currently employed?
- ○ Yes
- ○ No→ Go to #72

71. How far do you typically travel from your home to work (one-way)?
- ○ Less than 1 mile
- ○ 1 mile to less than 5 miles
- ○ 5 miles to less than 10 miles
- ○ 10 miles to less than 15 miles
- ○ 15 miles to less than 30 miles
- ○ 30 or more miles

72. How far do you typically travel one-way from your home for each of the following...?

	Less than 1 mile	1 mile to less than 5 miles	5 miles to less than 10 miles	10 miles to less than 15 miles	15 miles to less than 30 miles	30 or more miles
a. To shop for your regular household needs (e.g., groceries, clothing, or other household supplies)	○	○	○	○	○	○
b. For regular (non-emergency) medical, dental, pharmacy, or other health-related services	○	○	○	○	○	○
c. To receive medical care for a really bad emergency	○	○	○	○	○	○
d. To receive medical care for a less serious emergency	○	○	○	○	○	○

73. On average, how much time do you currently spend traveling (one-way) for medical, dental, or other health care appointments?
- ○ Less than 5 minutes
- ○ 5-9 minutes
- ○ 10-19 minutes
- ○ 20-29 minutes
- ○ 30 or more minutes

74. In the past 6 months, how many trips did you make for medical appointments (for yourself or others) that were at least 50 miles (one-way) from your home?

☐☐ trips

75. In the past 2 months, how many trips did you make that were at least 50 miles (one-way) from your home (for any reason)?

☐☐ trips

Demographics

76. In what Nebraska county do you currently live?

☐

77. What is the ZIP Code where you currently live?

☐☐☐☐☐

78. In what year were you born?

☐☐☐☐

79. Are you...
- ○ Male
- ○ Female
- ○ ☐

80. Are you of Hispanic (Latino/a) origin?
- ○ Yes
- ○ No

81. What is your race? *Mark all that apply.*
- ☐ White
- ☐ Black/African-American
- ☐ American Indian or Alaska Native
- ☐ Asian/Pacific Islander/Native Hawaiian
- ☐ I prefer not to answer

82. What is your martial status? *Mark only ONE.*
- ○ Married or living with a partner (cohabiting)
- ○ Divorced or Separated
- ○ Widowed
- ○ Single (never married, not living with partner)

83. What is the highest grade or level of school you completed?
- ○ Less than high school graduate
- ○ High school diploma or GED
- ○ Some college or Technical, Vocational, Associate, Junior College (2 yr, LPN, etc.)
- ○ Bachelor's degree or above (4 yr, BA, BS, RN, Masters, PhD, Law, Medicine)

84. Are you currently... *(check all that apply)*
- ☐ Homemaker
- ☐ Student
- ☐ Retired
- ☐ Employed for wages (full or part-time)
- ☐ Self-employed
- ☐ Out of work for less than 1 year
- ☐ Out of work for 1 year or more
- ☐ Disabled, unable to work
- ☐ Other (please specify) ☐

85. Have you ever served in the U.S. Armed Forces, Reserves or National Guard?
- ○ Yes
- ○ No

86. Were you born in the United States?
- ○ Yes → Go to #88
- ○ No

87. What year did you come to live in the United States?

☐☐☐☐

88. What is your primary language used in your home?
- ○ English
- ○ Spanish
- ○ Other (please specify) ☐

89. Including yourself, how many members of your household are 19 years of age or older?

☐☐ Adult(s)

90. Including yourself, how many adults age 65 or older live in your household?

☐☐ Adult(s)

91. How many children age 18 or less live in your household?

☐☐ Children

92. What is your annual household income (pre-tax), from all sources, in the past year?
- ○ $0 to $9,999
- ○ $10,000 to $19,999
- ○ $20,000 to $29,999
- ○ $30,000 to $39,999
- ○ $40,000 to $49,999
- ○ $50,000 to $74,999
- ○ $75,000 or more

Paper - Spanish

Salud y Acceso a Servicios Sanitarios en Nebraska

Estado de Salud

1. En general, diría que su estado de salud es...
   - ○ Excelente
   - ○ Muy Bueno
   - ○ Bueno
   - ○ Normal
   - ○ Malo

2. Si piensa en su salud física, incluyendo enfermedades físicas o lesiones, ¿en los últimos 30 días, en cuántos días su bienestar físico no fue bueno?

   [|] días

3. En cuanto a su salud mental, incluyendo estrés, depresión y problemas emocionales, ¿en los últimos 30 días, en cuántos días su bienestar físico no fue bueno?

   [|] días

4. Durante los últimos 30 días, ¿cuántos días su malestar físico o mental le impidió realizar actividades cotidianas, como cuidados personales, trabajar o actividades de ocio?

   [|] días

5. ¿Algún médico u otro profesional de la salud le ha dicho alguna vez que tiene alguno de estos problemas de salud?

	Yes	No
a. COVID-19 (coronavirus)	○	○
b. Problema cardíaco (ataque al corazón, cardiopatía, infarto de miocardio, angina, insuficiencia cardíaca congestiva)	○	○
c. Presión sanguínea alta (hipertensión)	○	○
d. Diabetes, prediabetes, diabetes limítrofe, o alto azúcar en sangre	○	○
e. Enfermedad pulmonar (EPOC, enfermedad pulmonar crónica, enfisema, bronquitis crónica, o asma)	○	○
f. Artritis (incluyendo osteoartritis o artritis reumatoide) o reumatismo	○	○
g. Derrame cerebral	○	○
h. Cáncer (por favor, especifique abajo)	○	○
[ ]		
i. Depresión o ansiedad	○	○

6. En los últimos 12 meses, ¿ha tenido que pasar alguna noche hospitalizado?
   - ○ Sí
   - ○ No

7. ¿Alguna vez ha tenido alguno de los siguientes tipos de cirugía?

	Sí	No
a. Reparación o reemplazo de rodilla	○	○
b. Reparación o reemplazo de cadera	○	○
c. Cirugía en la espalda o espina	○	○
d. Cirugía cardíaca de cualquier tipo, incluyendo baipás, (*bypass*), cirugía valvular, o el implante de una férula	○	○

8. ¿Cuánta dificultad tiene para caminar o subir escaleras?
   - ○ Sin dificultad
   - ○ Alguna dificultad
   - ○ Mucha dificultad
   - ○ Resulta imposible

Hábitos de Salud

9. Durante el pasado mes, además de su trabajo habitual, ¿ha realizado alguna actividad física o ejercicio? *Por ejemplo, correr, calistenia, golf, jardinería, o caminar como ejercicio.*
   - ○ Sí
   - ○ No

10. En los últimos 12 meses ¿ha recibido alguna dosis o vacuna contra la gripe que se aplique –rocíe- en la nariz?
    - ○ Sí
    - ○ No

11. ¿Ha fumado al menos 100 cigarros (5 paquetes de tabaco) en toda su vida?
    - ○ Sí
    - ○ No

12. ¿Alguna vez ha usado un cigarrillo electrónico u otro tipo de dispositivos vaporizadores (como un *e-cigarette*, vape, Juul, NJOY, Blu), aunque fuera una vez en su vida?
    - ○ Sí
    - ○ No

13. Durante los últimos 30 días, ¿cuántos días tomó al menos una bebida alcohólica, como cerveza, vino, una bebida de malta o licor?

    [|] días

Las siguientes preguntas tratan de examinaciones para la detección de cáncer…

14. Una prueba de sangre en las heces es una prueba que se realiza con un kit especial en casa, la cual determina si las heces contienen sangre. ¿Alguna vez se ha hecho esta prueba?
   - ◯ Sí
   - ◯ No→ Vaya a #16

15. ¿Cuándo fue su análisis de sangre en heces más reciente?

   Mes ☐☐ Año ☐☐☐☐

16. La sigmoidoscopia y la colonoscopia son exámenes en los que se inserta un tubo en el recto para observar el colon en busca de indicios de cáncer u otros problemas de salud. ¿Alguna vez ha tenido alguno de estos análisis?
   - ◯ Sí
   - ◯ No→ Vaya a #18

17. ¿Cuándo se realizó su última sigmoidoscopia o colonoscopia?

   Mes ☐☐ Año ☐☐☐☐

18. ¿Alguna vez le han hecho pruebas para detectar cáncer de pulmón?
   - ◯ Sí
   - ◯ No→ Vaya a #20

19. ¿Cuándo fue su último examen por cáncer de pulmón?

   Mes ☐☐ Año ☐☐☐☐

20. Para las mujeres: ¿alguna vez se ha hecho una mamografía?
   - ◯ Sí
   - ◯ No→ Vaya a #22

21. ¿Cuándo se realizó su última mamografía?

   Mes ☐☐ Año ☐☐☐☐

22. Para las mujeres, alguna vez se ha hecho la prueba de Papanicolaou (también llamada citología vaginal)?
   - ◯ Sí
   - ◯ No→ Vaya a #24

23. ¿Cuándo fue su última prueba de Papanicolaou (también llamada citología vaginal)?

   Mes ☐☐ Año ☐☐☐☐

24. Para los hombres: ¿alguna vez le han examinado en busca de cáncer de próstata (a través de un antígeno específico prostático o un test APE)?
   - ◯ Sí
   - ◯ No→ Vaya a #26

25. ¿Cuándo fue su examen de próstata más reciente?

   Mes ☐☐ Año ☐☐☐☐

Acceso al Sistema Sanitario y su Utilización

26. ¿Tiene actualmente alguno de los siguientes tipos de seguro médico o cobertura médica?

	Sí	No
a. Seguro médico a través de un actual o anterior empleador o sindicato	◯	◯
b. Seguro médico adquirido directamente de una compañía de seguros	◯	◯
c. Medicare (para personas con al menos 65 años de edad, o que padezcan determinadas discapacidades)	◯	◯
d. Medicaid, Asistencia Médica (o cualquier tipo del plan asistido por el gobierno para aquellas personas con bajos ingresos o discapacidad)	◯	◯
e. TRICARE (CHAMPUS) u otro servicio de salud militar	◯	◯
f. VA (incluidos aquellos que alguna vez han usado o se han inscrito en el cuidado de la salud de VA)	◯	◯
g. Seguro médico para nativos americanos	◯	◯
h. Cualquier otro tipo de seguro médico o plan de cobertura para su salud (por favor especifique el tipo) ☐	◯	◯
i. No tengo seguro médico	◯	◯

27. Si actualmente <u>no tiene</u> una cobertura médica, ¿aproximadamente cuándo fue la última vez que la tuvo?

   Mes ☐☐ Año ☐☐☐☐

28. Los navegadores de seguros informan a los usuarios acerca de los planes de servicios médicos, los créditos fiscales y la distribución de los costes. Además, ayudan a las personas a inscribirse en planes de salud con prestaciones disponibles en los seguros médicos del mercado. ¿Alguna vez ha usado un navegador de seguros?
- ◯ Sí
- ◯ No

29. Un navegador para pacientes o un defensor de pacientes guía al paciente a través del sistema médico y lo ayuda a comunicarse con los proveedores sanitarios. El navegador para pacientes también funciona para superar los obstáculos que se interponen en el acceso del paciente a la recepción de asistencia médica. ¿Alguna vez ha usado un navegador para pacientes o un coordinador de cuidados para pacientes?
- ◯ Sí
- ◯ No

30. *Telehealth* ('tele-salud') es un término amplio que se refiere al suministro de educación sanitaria y servicios médicos a través telecomunicación. Incluye la monitorización remota de constantes vitales, consultas, evaluaciones, diagnósticos y prescripciones. ¿Alguna vez ha usado *telehealth*?
- ◯ Sí
- ◯ No

31. ¿Tiene una persona que considere su médico personal o su proveedor de atención médica?
- ◯ Sí, solo una
- ◯ Sí, más de una
- ◯ No, no tengo una persona a la que considere mi médico personal o mi proveedor de atención médica.

32. ¿Tiene en la actualidad alguna factura de servicios sanitarios que se están pagando pasado el plazo límite?
- ◯ Sí
- ◯ No

33. ¿En algún momento <u>en los últimos 12 meses</u> ha necesitado una visita médica pero no pudo hacerla por el coste?
- ◯ Sí
- ◯ No

34. <u>Aparte del coste,</u> ¿en algún momento en los últimos 12 meses ha retrasado una visita médica por alguna de las siguientes razones?

	Sí	No
a. No pudo contactar por teléfono (línea ocupada, le pusieron en espera durante mucho tiempo, nadie respondió)	◯	◯
b. No pudo recibir una cita a tiempo	◯	◯
c. Una vez allí, tuvo que esperar mucho para ser atendido por un doctor	◯	◯
d. La clínica no estaba abierta cuando llegó	◯	◯
e. No tenía transporte	◯	◯
f. Tenía que cuidar a otro familiar necesitado (niños, ancianos, etc.)	◯	◯
g. No pudo tomarse el día en el trabajo	◯	◯
h. Tuvo problemas en la comunicación debido al idioma	◯	◯

35. <u>Además de en los últimos 12 meses</u>, ¿alguna vez tuvo que posponer recibir asistencia médica porque tenía preocupaciones acerca del coste?
- ◯ Sí
- ◯ No

36. <u>Además de en los últimos 12 meses</u>, ¿alguna vez tuvo que posponer recibir asistencia médica por la distancia o problemas con el transporte?
- ◯ Sí
- ◯ No

37. Sin incluir los medicamentos sin receta médica, ¿alguna vez <u>en los últimos 12 meses</u> no tomó su medicación como se le prescribió debido al coste?
- ◯ Sí
- ◯ No
- ◯ No se aplica (no le recetaron ningún medicamento)

38. ¿Alguna vez tuvo dificultades para obtener una receta médica debido a que no tenía un medio de transporte fiable?
- ◯ Sí
- ◯ No
- ◯ No se aplica (no le recetaron ningún medicamento)

39. Una revisión rutinaria es un chequeo general físico, no un examen para una condición médica, enfermedad o lesión específica. ¿Cuánto tiempo ha pasado desde que tuvo una revisión rutinaria hecha por un médico u otro profesional de salud por última vez?
- ◯ Menos de 12 meses
- ◯ Entre uno y menos de dos años
- ◯ Entre 2 y menos de 3 años
- ◯ Entre 3 y menos de 5 años
- ◯ 5 años o más
- ◯ Nunca

40. En los últimos 12 meses, ¿cuántas veces ha visitado a un profesional de salud por...

a. Una condición crónica (como artritis, diabetes, enfermedad cardíaca, cáncer o asma)?	☐	Número de visitas
b. Una afección grave (como un ataque al corazón, un hueso roto, una lesión, fiebre súbita, dolores de pecho agudos, ataques de asma graves)?	☐	Número de visitas
c. Salud mental o de comportamiento?	☐	Número de visitas

41. En los últimos 12 meses, ¿cuántas citas ha tenido en relación a un cáncer (exámenes, diagnósticos, tratamientos, o seguimiento)?

☐ Número de visitas

42. En los últimos 12 meses, ¿cuántas veces ha estado en la sala de emergencias?

☐ Número de visitas

43. En los últimos 12 meses, ¿cuántas veces ha acompañado a otra persona a una cita médica o a una visita a un profesional de salud?

☐ Número de visitas

44. En los últimos 12 meses, ¿cuántas veces ha acompañado a otra persona a una cita relacionada con cáncer (exámenes, diagnósticos, tratamientos, o seguimiento)?

☐ Número de visitas

45. Incluyendo todo tipo de dentistas (higienista dental, dentista, ortodoncista, cirujano dental y cualquier otro tipo de especialista dental), ¿cuánto tiempo hace que vistió por última vez un dentista o una clínica dental por cualquier motivo?

- ○ Menos de 12 meses
- ○ Entre 1 y menos de 2 años
- ○ Entre 2 y menos de 3 años
- ○ Entre 3 y menos de 5 años
- ○ 5 años o más
- ○ Nunca

Lista de problemas

46. Por favor, indique si alguna circunstancia de las siguientes ha sido un problema para usted en los últimos 30 días.

	Sí	No
a. Cuidado de niños	○	○
b. Cuidado de un adulto dependiente	○	○
c. Incapaz de trabajar debido a una discapacidad	○	○
d. Un niño con necesidades de educación especial insatisfechas	○	○
e. Jornadas laborales reducidas (incluyendo permisos o despidos)	○	○
f. Seguro médico	○	○
g. Prestaciones por desempleo	○	○
h. Prestaciones por discapacidad	○	○
i. SNAP/WIC (programas públicos de salud y nutrición)	○	○
j. Quedarse sin comida antes de poder comprar más	○	○
k. No se puede permitir por razones económicas una dieta equilibrada	○	○
l. Insectos o roedores en la vivienda	○	○
m. Pintura con plomo en la vivienda	○	○
n. Moho o humedades en la vivienda	○	○
o. Amenaza de desahucio	○	○
p. Servicios inestables	○	○
q. Limpieza general de la vivienda	○	○
r. Hacinamiento o saturación en la vivienda	○	○
s. Transporte	○	○
t. Cuidados médicos (para usted u otro miembro)	○	○
u. Estatus legal (preocupación por la situación de inmigración de la familia)	○	○
v. Estabilidad personal y familiar (por ejemplo, violencia doméstica, conflictos de tutela o custodia, bienestar de los niños)	○	○
w. Otros (por favor, especifique)	○	○

47. Por favor, indique si alguna de las siguientes circunstancias ha sido un problema para usted durante 2019.

	Sí	No
a. Cuidado de niños	○	○
b. Cuidado de un adulto dependiente	○	○
c. Incapaz de trabajar debido a una discapacidad	○	○
d. Un niño con necesidades de educación especial insatisfechas	○	○
e. Jornadas laborales reducidas (incluyendo permisos o despidos)	○	○
f. Seguro médico	○	○
g. Prestaciones por desempleo	○	○
h. Prestaciones por discapacidad	○	○
i. SNAP/WIC (programas públicos de salud y nutrición)	○	○
j. Quedarse sin comida antes de poder comprar más	○	○
k. No se puede permitir por razones económicas una dieta equilibrada	○	○
l. Insectos o roedores en la vivienda	○	○
m. Pintura con plomo en la vivienda	○	○
n. Moho o humedades en la vivienda	○	○
o. Amenaza de desahucio	○	○
p. Servicios inestables	○	○
q. Limpieza general de la vivienda	○	○
r. Hacinamiento o saturación en la vivienda	○	○
s. Transporte	○	○
t. Cuidados médicos (para usted u otro miembro)	○	○
u. Estatus legal (preocupación por la situación de inmigración de la familia)	○	○
v. Estabilidad personal y familiar (por ejemplo, violencia doméstica, conflictos de tutela o custodia, bienestar de los niños)	○	○
w. Otros (por favor, especifique) ______	○	○

Acceso al Teléfono y a la Computadora

48. ¿Posee o utiliza usted o algún miembro de su vivienda alguno de los siguientes tipos de computadoras?

	Sí	No
a. Computadora de mesa, computadora portátil, *tablet* u otro tipo de computadora inalámbrica?	○	○
b. *Smartphone*	○	○

49. ¿Tiene usted o algún miembro de su vivienda acceso a internet?

○ Sí
○ No→ **Vaya a #51**

50. ¿Tiene usted o algún miembro de su vivienda acceso a internet usando alguno de los dispositivos siguientes?

	Sí	No
a. Plan de datos móviles para un *smartphone* u otro tipo de dispositivo móvil	○	○
b. Servicio de internet de banda ancha (alta velocidad) como cables, fibra óptica o servicio DSL instalado en su casa	○	○
c. Servicio de internet por satélite instalado en su casa	○	○
d. Servicio de internet por línea conmutada instalada en su casa	○	○

51. ¿Cómo es la señal telefónica en su casa?

○ Buena
○ Suficiente
○ Mala

Posesión de Vehículos

52. ¿Cuántos vehículos posee usted o los miembros de su vivienda? Incluyendo carros, camiones, todoterrenos o furgonetas. ***No incluya vehículos recreativos como motocicletas, tráilers o remolques, autocaravanas o casas sobre ruedas, ATV, motonieves, barcos o aviones.***

[|] vehículos

53. ¿Cuántos vehículos funcionan y están disponibles para el uso de los miembros en este momento?

[|] vehículos

54. ¿Posee usted o alguno de los miembros de su vivienda alguna motocicleta?

○ Sí
○ No → **Vaya a #57**

55. ¿Cuántas motocicletas funcionan y están disponibles para el uso de los miembros en este momento?

[|] vehículos

56. ¿Usted utiliza la moto principalmente para...?

○ Ocio
○ Desplazarse al trabajo o la escuela
○ Hacer mandados/recados

Transporte

57. ¿Tiene licencia válida para conducir?

○ Sí
○ No

58. ¿Tiene alguien en su vivienda un carné o licencia para conducir válida?

○ Sí
○ No

59. ¿ Alguna vez ha dejado un trabajo o lo ha perdido porque no tenía un medio de transporte de confianza para llegar?
- ◯ Sí
- ◯ No

60. ¿La falta de opciones de transporte le impide ir al trabajo ahora?
- ◯ Sí
- ◯ No

61. ¿Cuál es la cantidad máxima que estaría dispuesto a pagar por un viaje de ida desde o hacia su lugar de trabajo?
- ◯ Menos de un dólar
- ◯ $1.00 - $1.99
- ◯ $2.00 - $2.99
- ◯ $3.00 o más

62. ¿Cuál es la cantidad máxima que estaría dispuesto a pagar por un viaje de ida desde o hacia un servicio o cita médica?
- ◯ Menos de un dólar
- ◯ $1.00 - $1.99
- ◯ $2.00 - $2.99
- ◯ $3.00 o más

63. Si tiene que ir a algún sitio en carro, ¿qué dificultad tiene en llegar allí?
- ◯ Mucha dificultad
- ◯ Alguna dificultad
- ◯ Sin dificultad

64. El transporte médico no urgente (NEMT) es un servicio para los beneficiarios de *Medicaid* que cualifican por necesidades de transporte. Ofrece transporte para las citas no urgentes pero médicamente necesarias. ¿Alguna vez ha usado NEMT (para usted o para acompañar a alguien)?
- ◯ Sí
- ◯ No→ Vaya a #66

65. Cuando utilizó NEMT, ¿experimentó alguna de las siguientes circunstancias?

	Sí	No
a. El vehículo llevaba a otros pasajeros al mismo tiempo	◯	◯
b. Hubo dificultad para programar un viaje (hacer una reserva)	◯	◯
c. El vehículo llegó tarde	◯	◯
d. El vehículo no apareció	◯	◯
e. El vehículo no pudo acomodar las necesidades físicas (como la accesibilidad para sillas de ruedas)	◯	◯
f. El recorrido tomó demasiado tiempo porque tuvo que hacer otras paradas	◯	◯

66. ¿Utiliza alguno de los siguientes medios para llegar a sus citas médicas?

	Sí	No
a. Conduce usted	◯	◯
b. Le lleva algún miembro de su familia o un amigo	◯	◯
c. Camina (o va en silla de ruedas)	◯	◯
d. Va en bicicleta	◯	◯
e. Toma el transporte público (autobús, tren, metro)	◯	◯
f. Toma un taxi	◯	◯
g. Usa una plataforma de transporte compartido de pago como Uber o Lyft	◯	◯
h. Usa una furgoneta o un autobús de transporte proporcionado por su lugar de residencia	◯	◯
i. Usa una furgoneta o autobús lanzadera para personas mayores o personas con discapacidad	◯	◯
j. Usa transporte ofrecido por el centro médico (como un carro o furgoneta, o un transporte para no emergencias)	◯	◯
k. Otro (por favor, especifique)	◯	◯

67. ¿Hay algún pariente o amigo que normalmente transporte a uno de los miembros de su hogar?
- ◯ Sí
- ◯ No

68. Durante el último año, ¿tuvo algún problema con el transporte que le impidió hacer lo siguiente?

	Sí	No
a. Visitar a amigos o familiares que no viven con usted	◯	◯
b. Asistir a servicios religiosos	◯	◯
c. Participar en clubs, clases, u otras actividades organizadas	◯	◯
d. Realizar salidas de ocio (como salir a comer, apostar, escuchar música, ver una película, ir a un partido o un evento deportivo)	◯	◯
e. Solicitar asistencia médica	◯	◯

69. En los <u>últimos 30 días,</u> ¿con qué frecuencia utilizó alguno de los siguientes medios de transporte para llegar a destinos fuera de su vivienda?

	7 días a la semana	5 - 6 días a la semana	2 - 4 días a la semana	Una vez por semana o menos	Nunca
a. Condujo usted en un vehículo personal (carro, todoterreno, furgoneta, camioneta, moto)	○	○	○	○	○
b. Caminó (o fue en silla de ruedas)	○	○	○	○	○
c. Fue en bicicleta	○	○	○	○	○
d. Le transportó un miembro de su familia o un amigo	○	○	○	○	○
e. Tomó transporte público (autobús, tren, metro o subterráneo)	○	○	○	○	○
f. Tomó un taxi	○	○	○	○	○
g. Usó una plataforma de servicio compartido de pago como Uber o Lyft	○	○	○	○	○
h. Usó una furgoneta o un autobús de transporte proporcionado por su lugar de residencia	○	○	○	○	○
i. Usó una furgoneta o autobús lanzadera para personas mayores o personas discapacitadas	○	○	○	○	○
j. Condujo un carrito de golf, Segway, o un cortacésped.	○	○	○	○	○
k. Condujo maquinaria agrícola	○	○	○	○	○
l. Montó un caballo, burro o asno o condujo un carruaje tirado por caballos	○	○	○	○	○

Distancia de los trayectos

70. ¿Actualmente, tiene usted trabajo?
- ○ Sí
- ○ No→ Vaya a #72

71. ¿Cuánta distancia debe recorrer normalmente de casa al trabajo (<u>solo de ida</u>)?
- ○ Menos de una milla
- ○ Entre 1 y menos de 5 millas
- ○ Entre 5 y menos de 10 millas
- ○ Entre 10 y menos de 15 millas
- ○ Entre 15 y menos de 30 millas
- ○ 30 o más milla

72. ¿Cuánta distancia recorre <u>de ida</u> normalmente desde su casa por cada una de las siguientes razones?

	Menos de una milla	De una a menos de 5 millas	De 5 a menos de 10 millas	De 10 a menos de 15 millas	De 15 a menos de 30 millas	30 millas o más
a. Para comprar productos de necesidad básica para su hogar (comida, ropa, u otros productos domésticos).	○	○	○	○	○	○
b. Para servicios regulares (no urgentes) médicos, dentales, farmacéuticos, u otro tipo de servicios de salud.	○	○	○	○	○	○
c. Para recibir asistencia médica por una emergencia <u>muy grave.</u>	○	○	○	○	○	○
d. Para recibir asistencia médica por una emergencia <u>menos grave.</u>	○	○	○	○	○	○

73. De media, ¿cuánto tiempo le toma desplazarse (solo de ida) para citas médicas, dentales, u otro tipo de servicios de salud?
- ○ Menos de 5 minutos
- ○ De 5 a 9 minutos
- ○ De 10 a 19 minutos
- ○ De 20 a 29 minutos
- ○ 30 minutos o más

74. En los últimos 6 meses, ¿cuántos viajes realizó para citas médicas (para usted o para otros) que estaban al menos a 50 millas de distancia (solo de ida) desde su casa?

☐☐ viajes

75. En los últimos 2 meses, ¿cuántos viajes realizó que fueran al menos de 50 millas (solo de ida) desde su casa (por cualquier motivo)?

☐☐ viajes

Demografía

76. ¿En qué condado de Nebraska reside actualmente?

☐

77. ¿Cuál es el código postal de su residencia?

☐☐☐☐☐

78. ¿En qué año nació?

☐☐☐☐

79. ¿Es usted...?
- ○ Hombre
- ○ Mujer
- ○ ☐

80. ¿Es usted de origen hispano (latino/a)?
- ○ Sí
- ○ No

81. ¿Cuál es su raza? *(seleccione todas las casillas que aplican).*
- ☐ Blanco
- ☐ Negro/Afro-americano
- ☐ Nativo americano o nativo de Alaska
- ☐ Asiático/Isleño del Pacífico/Nativo hawaiano
- ☐ Prefiero no contestar

82. ¿Cuál es su estado civil? *Marque solo UNA casilla.*
- ○ Casado/a o viviendo con una pareja (cohabitando)
- ○ Divorciado/a o separado/a
- ○ Viudo/a
- ○ Soltero/a (nunca se ha casado, ni vive con una pareja)

83. ¿Cuál es el nivel más alto de educación que usted ha completado?
- ○ Menos de graduado de la secundaria
- ○ Diploma de escuela secundaria o GED
- ○ Algún colegiado o grado técnico, vocacional, asociado, o grado de iniciación (2 años, LPN, etc.)
- ○ Grado universitario o superior (4 años, BA, BS, RN, máster(s), doctorado, Derecho, Medicina)

84. En la actualidad, usted... *(seleccione todas las casillas que aplican)*
- ☐ Es amo/a de casa
- ☐ Es estudiante
- ☐ Está jubilado
- ☐ Es empleado remunerado (a jornada completa o parcial)
- ☐ Es autónomo
- ☐ Lleva desempleado menos de un año
- ☐ Lleva desempleado un año o más
- ☐ No puede trabajar por alguna discapacidad
- ☐ Otro (por favor, especifique) ☐

85. ¿Alguna vez ha servido en las Fuerzas Armadas estadounidenses, Reservas o en la Guardia Nacional?
- ○ Sí
- ○ No

86. ¿Nació en los Estados Unidos?
- ○ Sí → Vaya a #88
- ○ No

87. ¿En qué año vino a vivir a los Estados Unidos?

☐☐☐☐

88. ¿Qué lengua utiliza principalmente en su hogar?
- ○ Inglés
- ○ Español
- ○ Otra (por favor, especifique) ☐

89. Incluyéndose a usted, ¿cuántos miembros de su unidad doméstica tienen 19 años o más?

☐☐ Adulto(s)

90. Incluyéndose a usted, ¿cuántos adultos de 65 años o más viven en su hogar?

☐☐ Adulto(s)

91. ¿Cuántas personas de 18 años o menos viven en su hogar?

☐☐ Personas menores

92. ¿Cuál es el salario anual de su unidad doméstica (antes de impuestos), de todas las fuentes de ingresos, en este último año?
- ○ De $0 a $9,999
- ○ De $10,000 a $19,999
- ○ De $20,000 a $29,999
- ○ De $30,000 a $39,999
- ○ De $40,000 a $49,999
- ○ De $50,000 a $74,999
- ○ $75,000 o más

Web - English

English

Welcome to the Health and Health Care Access in Nebraska Survey. If you would like to take the survey in Spanish you can change the language using the drop down menu in the upper right hand corner. Thank you again for your participation!

Bienvenido al Acceso de Salud y Cuidado de la Salud de la Encuesta de Nebraska. Si desea tomar el formulario en español, usted puede cambiar el idioma usando el menú desplegable que se encuentra en la parte de arriba en la esquina a la derecha. Nuevamente, gracias por su participación!

○ Continue to survey

Health Status

Would you say that in general your health is...

- ○ Excellent
- ○ Very good
- ○ Good
- ○ Fair
- ○ Poor

Thinking about your physical health, which includes physical illness and injury, for how many days during the past 30 days were your physical health NOT good?

[ ]

Thinking about your mental health, which includes stress, depression, and problems with emotions, for how many days during the past 30 days was your mental health NOT good?

[ ]

During the past 30 days, for how many days did poor physical or mental health keep you from doing your usual activities, such as self-care, work, or recreation?

[ ]

English

Has a doctor or other health professional ever told you that you had any of the following medical conditions?

	Yes	No
COVID-19 (coronavirus)	O	O
Heart condition (heart attack, heart disease, myocardial infraction, angina, congestive heart failure)	O	O
High blood pressure (hypertension)	O	O
Diabetes, pre-diabetes, borderline diabetes, or high blood sugar	O	O
Lung disease (COPD, chronic lung disease, emphysema, chronic bronchitis, or asthma)	O	O
Arthritis (including osteo or rheumatoid arthritis) or rheumatism	O	O
Stroke	O	O
Cancer	O	O
Depression or anxiety disorder	O	O

*If they selected to yes to Cancer

You indicated that a doctor or other health professional told you that you had cancer. Can you please specify the type of cancer that it was?

English

In the past 12 months, have you had an overnight hospital stay?

- ○ Yes
- ○ No

Have you ever had any of the following types of surgery?

	Yes	No
Knee repair or replacement surgery	○	○
Hip repair or replacement surgery	○	○
Back or spine surgery	○	○
Heart surgery of any kind, including a bypass, valve surgery, or stent	○	○

How much difficulty do you have walking or climbing steps?

- ○ No difficulty
- ○ Some difficulty
- ○ A lot of difficulty
- ○ Cannot do at all

Health Behaviors

During the past month, other than your regular job, did you participate in any physical activities or exercises? *Examples might include running, calisthenics, golf, gardening, or walking for exercise.*

- ○ Yes
- ○ No

During the past 12 months, have you had either a flu shot or a flu vaccine that was sprayed in your nose?

- ○ Yes
- ○ No

Have you smoked at least 100 cigarettes (5 packs) in your lifetime?

- ○ Yes
- ○ No

Have you ever used an electronic cigarette or other electronic vaping products (such as e-cigarette, vape, Juul, NJOY, Blu), even just once in your entire life?

- ○ Yes
- ○ No

During the past 30 days, how many days did you have at least one drink of any alcoholic beverage such as beer, wine, a malt beverage or liquor?

[ ]

The next questions are about cancer screening...

A blood stool test is a test that may use a special kit at home to determine whether the stool contains blood. Have you ever had this test?

- ○ Yes
- ○ No

*If they selected yes

When was your most recent blood stool test?

Month

Year

Sigmoidoscopy and colonoscopy are exams in which a tube is inserted in the rectum to view the colon for signs of cancer or other health problems. Have you ever had either of these exams?

- ○ Yes
- ○ No

*If they selected yes

When was your most recent sigmoidoscopy or colonoscopy?

Month

Year

Have you ever been screened for lung cancer?

- ○ Yes
- ○ No

*If they selected yes

When was your most recent lung cancer screening?

Month ☐

Year ☐

For women: Have you ever had a mammogram?

- ○ Yes
- ○ No

*If they selected yes

When was your most recent mammogram?

Month ☐

Year ☐

For women: Have you ever had a Pap test?

- ○ Yes
- ○ No

*If they selected yes

When was your most recent Pap test?

Month ☐

Year ☐

For men: Have you ever been screened for prostate cancer (such as the Prostate-Specific Antigen or PSA test)?

- ○ Yes
- ○ No

*If they selected yes

When was your most recent screening?

Month

Year

Health Care Access and Utilization

Are you currently covered by any of the following types of health insurance or health coverage plans?

	Yes	No
Insurance through a current or former employer or union	○	○
Insurance purchased directly from an insurance company (marketplace)	○	○
Medicare (for people 65 and older, or people with certain disabilities)	○	○
Medicaid, Medical Assistance, or any kind of government-assisted plan for those with low income or a disability	○	○
TRICARE (CHAMPUS) or other military health care	○	○
VA (including those who have ever used or enrolled in VA health care	○	○
Indian Health Service	○	○
Any other type of health insurance or health coverage plan	○	○
Do not have health insurance	○	○

*If they selected yes for any other type

You stated that you are currently covered by another type of health insurance or health coverage plan. Can you please specify what it is?

____

If you do not currently have health care coverage, about when did you last have coverage?

Month ______

Year ______

Insurance navigators educate the public about health care plans, tax credits, and cost sharing. They also help enroll people into health benefit plans on the health insurance marketplace. Have you ever used an insurance navigator?

- ○ Yes
- ○ No

A patient navigator or patient advocate helps guide a patient through the healthcare system and communicate with healthcare providers. A patient navigator also works to overcome obstacles that are in the way of the patient receiving care. Have you ever used a patient navigator or patient care coordinator?

- ○ Yes
- ○ No

Telehealth is a board term referring to provision of health education and medical services through telecommunications technology. It includes remote monitoring of vital signs, consultation, evaluation, diagnosis, and prescription. Have you ever used telehealth?

- ○ Yes
- ○ No

Do you have one person you think of as your personal doctor or health care provider?

- ○ Yes, only one
- ○ Yes, more than one
- ○ No, there is no person I think of as my personal doctor or health care provider

Do you currently have any health care bills that are being paid off over time?

- ○ Yes
- ○ No

Was there a time <u>in the past 12 months</u> when you needed to see a doctor but could not because of cost?

- ○ Yes
- ○ No

<u>Other than cost</u>, in the past 12 months have you delayed getting medical care for any of the following reasons?

	Yes	No
You couldn't get through on the phone (busy signal, you were put on a long hold, no one answered)	○	○
You couldn't get an appointment soon enough	○	○
Once you got there, you had to wait too long to see the doctor	○	○
The clinic or doctor's office wasn't open when you got there	○	○
You didn't have transportation	○	○
You didn't have care for another household member (childcare, eldercare)	○	○
You couldn't take time off from work	○	○
Language barriers	○	○

Other than the past 12 months, did you ever put off receiving health care because you were concerned about cost?

- ○ Yes
- ○ No

Other than the past 12 months, did you ever put off receiving health care because of distance or transportation concerns?

- ○ Yes
- ○ No

Not including over the counter medications, was there a time in the past 12 months, when you did not take your medications as prescribed because of cost?

- ○ Yes
- ○ No
- ○ Not applicable (no medication was prescribed)

Have you ever had difficulty filling a prescription because you had no reliable transportation?

- Yes
- No
- Not applicable (no medication was prescribed)

A routine checkup is a general physical exam, not an exam for a specific injury, illness, or condition. About how long has it been since you last saw a doctor or other health care professional for a routine checkup?

- Less than 12 months ago
- 1 year to less than 2 years ago
- 2 years to less than 3 years ago
- 3 years to less than 5 years ago
- 5 years or more
- Never

In the past 12 months, how many times have you seen a health professional for...

Chronic condition (such as arthritis, diabetes, heart disease, cancer or asthma)?	☐ Number of visits
Acute condition (such as heart attack, broken bone, injury, sudden fever, severe chest pains, severe asthma attack)?	☐ Number of visits
Mental or behavioral health?	☐ Number of visits

In the past 12 months, how many times have you been to a cancer-related appointment (screening, diagnosis, treatment, or follow-up)?

☐ Number of visits

In the past 12 months, how many times have you been to the emergency room?

☐ Number of visits

In the past 12 months, how many times have you accompanied someone else to a medical appointment or visit to a health professional?

[ ] Number of visits

In the past 12 months, how many times have you accompanied someone else to a cancer-related appointment (screening, diagnosis, treatment, or follow-up)?

[ ] Number of visits

Including all types of dentists (dental hygienist, dentist, orthodontist, oral surgeon, and all other dental specialists), how long has it been since you last visited a dentist or a dental clinic for any reason?

- Less than 12 months ago
- 1 year to less than 2 years ago
- 2 years to less than 3 years ago
- 3 years to less than 5 years ago
- 5 years ago or more
- Never

Problems List

Please indicate if any of the following has been a problem for you in the past 30 days.

	Yes	No
Child care	O	O
Dependent adult care	O	O
Unable to work due to disability	O	O
Child with unmet special education needs	O	O
Reduced work hours (including furlough or lay-off)	O	O
Health insurance	O	O
Unemployment benefits	O	O
Disability benefits	O	O
SNAP/WIC	O	O
Ran out of food before you could buy more	O	O
Couldn't afford to eat balanced meals	O	O
Bugs or rodents in household	O	O
Lead paint in household	O	O
Mold or dampness in household	O	O
Threat of eviction	O	O
Unreliable utilities	O	O
General cleanliness of household	O	O

Overcrowding in household	○	○
Transportation	○	○
Health care (for self or household member)	○	○
Legal status (concerns with family immigration status)	○	○
Personal and family stability (for example, domestic violence, guardianship or custody issues, child welfare)	○	○
Other	○	○

*If they selected yes to other

You indicated that you have had other problems in the past 30 days. Can you please specify what those problems were?

Please indicate if any of the following has been a problem for you during 2019.

	Yes	No
Child care	○	○
Dependent adult care	○	○
Unable to work due to disability	○	○
Child with unmet special education needs	○	○
Reduced work hours (including furlough or lay-off)	○	○
Health insurance	○	○
Unemployment benefits	○	○
Disability benefits	○	○
SNAP/WIC	○	○
Ran out of food before you could buy more	○	○
Couldn't afford to eat balanced meals	○	○
Bugs or rodents in household	○	○
Lead paint in household	○	○
Mold or dampness in household	○	○
Threat of eviction	○	○
Unreliable utilities	○	○
General cleanliness of household	○	○

Overcrowding in household	○	○
Transportation	○	○
Health care (for self or household member)	○	○
Legal status (concerns with family immigration status)	○	○
Personal and family stability (for example, domestic violence, guardianship or custody issues, child welfare)	○	○
Other	○	○

*If they selected yes to other

You indicated that you have had other problems during 2019. Can you please specify what those problems were?

Phone and Computer Access

Do you or any member of your household own or use any of the following types of computers?

	Yes	No
Desktop, laptop, tablet or other portable wireless computer	○	○
Smartphone	○	○

Do you or any member of your household have access to the internet?

- ○ Yes
- ○ No

How is the cell phone reception at your home?

- ○ Good
- ○ Fair
- ○ Poor

*If they answered yes to Do you or any member of your household have access to the internet?

Do you or any member of your household have access to the internet using each of the following...?

	Yes	No
Cellular data plan for a smartphone or other mobile device	○	○
Broadband (high speed) internet service such as cable, fiber optic, or DSL service installed at your house	○	○
Satellite internet service installed at your house	○	○
Dial-up internet service installed at your house	○	○

Vehicle Ownership

How many vehicles do you or your household members own? Include cars, trucks, SUVs, or vans. *Do NOT include recreational vehicles such as motorcycles, trailers, motor homes, ATV, snowmobiles, boats, or airplanes.*

[ ] vehicles

How many vehicles are currently working and available for household members to use?

[ ] vehicles

Do you or your household members own any motorcycles?

○ Yes
○ No

*If they answered yes to owning motorcycles

How many motorcycles are currently working and available for household members to use?

[ ] vehicles

You primarily use your motorcycle for...?

- ○ Recreation
- ○ Commuting to work or school
- ○ Running errands

Transportation

Do you have a valid driver's license?

- ○ Yes
- ○ No

Does anyone in your household have a valid driver's license?

- ○ Yes
- ○ No

Have you ever quit a job or lost a job because you had no reliable transportation to get to work?

- ○ Yes
- ○ No

Does a lack of transportation options prevent you now from working?

- ○ Yes
- ○ No

What is the maximum amount you would be willing to pay for a one-way ride to or from work?

- ○ Less than $1.00
- ○ $1.00-$1.99
- ○ $2.00-$2.99
- ○ $3.00 or more

What is the maximum amount you would be willing to pay for a one-way ride to or from a medical or health service appointment?

- ○ Less than $1.00
- ○ $1.00-$1.99
- ○ $2.00-$2.99
- ○ $3.00 or more

If you have to get somewhere in a car, how difficult is it for you to get there?

- ○ Very difficult
- ○ Somewhat difficult
- ○ Not at all difficult

Non-emergency medical transportation (NEMT) is a service for Medicaid beneficiaries who qualify due to transportation needs. It provides transportation to non-emergency but medically necessary appointments. Have you ever used NEMT (for yourself or accompanying someone else)?

- ○ Yes
- ○ No

*If they answer yes

Did you experience any of the following when using NEMT...

	Yes	No
Ride transported other passengers at the same time	○	○
Difficulty scheduling a ride (making reservations)	○	○
Ride was late	○	○
Ride did not show up	○	○
Ride could not accommodate your physical needs (such as wheelchair accessible)	○	○
Ride took too long because it had to make other stops	○	○

Do you usually use each of the following to travel to your medical or health care appointments?

	Yes	No
Drive yourself	○	○
Get a ride from a family member or friend	○	○
Walk (or wheelchair)	○	○
Bicycle	○	○
Take public transportation (bus, train, subway)	○	○
Take a taxi	○	○
Use a paid ride share service such as Uber or Lyft	○	○
Use a van or shuttle service provided by the place you live	○	○
Use a van or shuttle service for seniors or people with disabilities	○	○
Use transportation provided by the medical facility (such as a car or van, Non-Emergency Medical Transportation)	○	○
Other	○	○

*If they answer yes to other

You indicated that you use another type of transportation to travel to your medical or health care appointments. Can you please specify?

Do you have a relative or friend who regularly provides transportation to a member of your household?

- ○ Yes
- ○ No

In the past year, did a transportation problem ever keep you from doing the following...

	Yes	No
Visiting in person with friends or family not living with you	○	○
Attending religious services	○	○
Participating in clubs, classes, or other organized activities	○	○
Going out for enjoyment (such as going out to dinner, to gamble, to hear music, or to see a movie, play or sporting event)	○	○
Seeking medical care	○	○

In the past 30 days, how often did you use the following modes of transportation to get to destinations outside your home?

	7 days/week	5-6 days/week	2-4 days/week	Once a week or less	Never
Drive yourself in a personal vehicle (car, SUV, van, pickup truck, motorcycle)	○	○	○	○	○
Walk (or wheelchair)	○	○	○	○	○
Bicycle	○	○	○	○	○
Get a ride from a family member or friend	○	○	○	○	○
Take public transportation (bus, train, subway)	○	○	○	○	○
Take a taxi	○	○	○	○	○
Use a paid ride share service such as Uber or Lyft	○	○	○	○	○
Use a van or shuttle service provided by the place you live	○	○	○	○	○
Use a van or shuttle service for seniors or people with disabilities	○	○	○	○	○
Drive a golf car, Segway, or riding lawn mower	○	○	○	○	○
Drive farm equipment	○	○	○	○	○
Ride a horse, donkey or burro or drive a horse-drawn carriage	○	○	○	○	○

Travel Distance

Are you currently employed?

- ○ Yes
- ○ No

*If they answered yes

How far do you typically travel from your home to work (one-way)?

- ◯ Less than 1 mile
- ◯ 1 mile to less than 5 miles
- ◯ 5 miles to less than 10 miles
- ◯ 10 miles to less than 15 miles
- ◯ 15 miles to less than 30 miles
- ◯ 30 or more miles

How far do you typically travel one-way from your home for each of the following...?

	Less than 1 mile	1 mile to less than 5 miles	5 miles to less than 10 miles	10 miles to less than 15 miles	15 miles to less than 30 miles	30 or more miles
To shop for your regular household needs (e.g., groceries, clothing, or other household supplies)	◯	◯	◯	◯	◯	◯
For regular (non-emergency) medical, dental, pharmacy, or other health-related services	◯	◯	◯	◯	◯	◯
To receive medical care for a really bad emergency	◯	◯	◯	◯	◯	◯
To receive medical care for a less serious emergency	◯	◯	◯	◯	◯	◯

On average, how much time do you currently spend traveling (one-way) for medical, dental, or other health care appointments?

- ○ Less than 5 minutes
- ○ 5-9 minutes
- ○ 10-19 minutes
- ○ 20-29 minutes
- ○ 30 or more minutes

In the past 6 months, how many trips did you make for medical appointments (for yourself or others) that were at least 50 miles (one-way) from your home?

[ ] trips

In the past 2 months, how many trips did you make that were at least 50 miles (one-way) from your home (for any reason)?

[ ] trips

Demographics

In what Nebraska county do you currently live?

What is the ZIP Code where you currently live?

In what year were you born?

Are you...

- Male
- Female
-

Are you of Hispanic (Latino/a) origin?

- ○ Yes
- ○ No

What is your race? *(check all that apply)*

- ☐ White
- ☐ Black or African American
- ☐ American Indian or Alaska Native
- ☐ Asian/Pacific Islander/Native Hawaiian

- ☐ I prefer not to answer

What is your marital status? *Mark only ONE.*

- ○ Married or living with a partner (cohabiting)
- ○ Divorced or Separated
- ○ Widowed
- ○ Single (never married, not living with partner)

What is the highest grade or level of school you completed?

- ○ Less than high school graduate
- ○ High school diploma or GED
- ○ Some college or Technical, Vocational, Associate, Junior College (2 yr, LPN, etc.)
- ○ Bachelor's degree or above (4 yr, BA, BS, RN, Masters, PhD, Law, Medicine)

Are you currently... *(check all that apply)*

- ☐ Homemaker
- ☐ Student
- ☐ Retired
- ☐ Employed for wages (full or part-time)
- ☐ Self-employed
- ☐ Out of work for less than 1 year
- ☐ Out of work for 1 year or more
- ☐ Disabled, unable to work
- ☐ Other

*If they selected other

When asked what you currently are, you indicated other. Can you please specify?

Have you ever served in the U.S. Armed Forces, Reserves or National Guard?

- ○ Yes
- ○ No

Were you born in the United States?

- Yes
- No

*If they answered no

What year did you come to live in the United States?

What is your primary language used in your home?

- English
- Spanish
- Other

*If they answered other

You indicated that the primary language used in your home was something else. Can you please specify?

Including yourself, how many members of your household are 19 years of age or older?

[ ] Adult(s)

Including yourself, how many adults age 65 or older live in your household?

[ ] Adult(s)

How many children age 18 or less live in your household?

[ ] Children

What is your annual household income (pre-tax), from all sources, in the past year?

- $0 to $9,999
- $10,000 to $19,999
- $20,000 to $29,999
- $30,000 to $39,999
- $40,000 to $49,999
- $50,000 to $74,999
- $75,000 or more

We thank you for your time spent taking this survey.
Your response has been recorded.

Health and Health Care Access in Nebraska
Questions? 1-800-480-4549 or bosr@unl.edu

Web - Spanish

Español

Welcome to the Health and Health Care Access in Nebraska Survey. Please select the language that you would like to take the survey in. Thank you again for your participation!

Bienvenido al Acceso de Salud y Cuidado de la Salud de la Encuesta de Nebraska. Si desea tomar el formulario en español, usted puede cambiar el idioma usando el menú desplegable que se encuentra en la parte de arriba en la esquina a la derecha. Nuevamente, gracias por su participación!

○ Continuar al formulario

Estado de salud

En general, diría que su estado de salud es…

- ○ Excelente
- ○ Muy bien
- ○ Bueno
- ○ normal
- ○ malo

Si piensa en su salud física, incluyendo enfermedades físicas o lesiones, ¿en los últimos 30 días, en cuántos días su bienestar físico no fue bueno?

[ ]

En cuanto a su salud mental, incluyendo estrés, depresión y problemas emocionales, ¿ en los últimos 30 días, en cuántos días su bienestar físico no fue bueno?

[ ]

Durante los últimos 30 días, ¿cuántos días su malestar físico o mental le impidió realizar actividades cotidianas, como cuidados personales, trabajar o actividades de ocio?

[ ]

¿Algún médico u otro profesional de la salud le ha dicho alguna vez que tiene alguno de estos problemas de salud?

	Sí	No
COVID-19 (coronavirus)	○	○
Problema cardíaco (ataque al corazón, cardiopatía, infarto de miocardio, angina, insuficiencia cardíaca congestiva)	○	○
Presión sanguínea alta (hipertensión)	○	○
Diabetes, prediabetes, diabetes limítrofe, o alto azúcar en sangre	○	○
Enfermedad pulmonar (EPOC, enfermedad pulmonar crónica, enfisema, bronquitis crónica, o asma)	○	○
Artritis (incluyendo osteoartritis o artritis reumatoide) o reumatismo	○	○
Derrame cerebral	○	○
Cáncer	○	○
Depresión o ansiedad	○	○

*If they selected yes for cancer

Usted indicó que un doctor u otro profesional de la salud le dijo a usted que tenía cáncer. Podría usted por favor especificar que tipo de cáncer era?

En los últimos 12 meses, ¿ha tenido que pasar alguna noche hospitalizado?

- Sí
- No

¿Alguna vez ha tenido alguno de los siguientes tipos de cirugía?

	Sí	No
Reparación o reemplazo de rodilla	○	○
Reparación o reemplazo de cadera	○	○
Cirugía en la espalda o espina	○	○
Cirugía cardíaca de cualquier tipo, incluyendo baipás, (bypass), cirugía valvular, o el implante de una férula	○	○

¿Cuánta dificultad tiene para caminar o subir escaleras?

- Sin dificultad
- Alguna dificultad
- Mucha dificultad
- Resulta imposible

Hábitos de salud

Durante el último mes, además de su trabajo habitual, ¿ha realizado alguna actividad física o ejercicio? *Por ejemplo, correr, calistenia, golf, jardinería, o caminar como ejercicio.*

- Sí
- No

En los últimos 12 meses ¿ha recibido alguna dosis o vacuna contra la gripe que se aplique –rocíe- en la nariz?

- Sí
- No

¿Ha fumado al menos 100 cigarrillos (5 paquetes de tabaco) en toda su vida?

- Sí
- No

¿Alguna vez ha usado un cigarrillo electrónico u otro tipo de dispositivos vaporizadores (como un e-cigarette, vape, Juul, NJOY, Blu), aunque fuera una vez en su vida?

- Sí
- No

Durante los últimos 30 días, ¿cuántos días tomó al menos una bebida alcohólica, como cerveza, vino, una bebida de malta o licor?

Las siguientes preguntas tratan de examinaciones para la detección de cáncer…

Una prueba de sangre en las heces es una prueba que se realiza con un kit especial en casa, la cual determina si las heces contienen sangre. ¿Alguna vez se ha hecho esta prueba?

- Sí
- No

*If they answered yes

¿Cuándo fue su análisis de sangre en heces más reciente?

Mes ____

Año ____

La sigmoidoscopia y la colonoscopia son exámenes en los que se inserta un tubo en el recto para observar el colon en busca de indicios de cáncer u otros problemas de salud. ¿Alguna vez ha tenido alguno de estos análisis?

- Sí
- No

*If they answered yes

¿Cuándo se realizó su última sigmoidoscopia o colonoscopia?

Mes ____

Año ____

¿Alguna vez le han hecho pruebas para detectar cáncer de pulmón?

- Sí
- No

*If they answered yes

¿Cuándo fue su último examen por cáncer de pulmón?

Mes

Año

Para las mujeres: ¿alguna vez se ha hecho una mamografía?

- Sí
- No

*If they answered yes

¿Cuándo se realizó su última mamografía?

Mes

Año

Para las mujeres, alguna vez se ha hecho la prueba de Papanicolaou (también llamada citología vaginal)?

- Sí
- No

*If they answered yes

¿Cuándo fue su última prueba de Papanicolaou (también llamada citología vaginal)?

Mes

Año

Para los hombres: ¿alguna vez le han examinado en busca de cáncer de próstata (a través de un antígeno específico prostático o un test APE)?

- Sí
- No

*If they answered yes

¿Cuándo fue su examen de próstata más reciente?

Mes

Año

Acceso al sistema sanitario y su utilización

¿Tiene actualmente alguno de los siguientes tipos de seguro médico o cobertura médica?

	Sí	No
Seguro médico a través de un actual o anterior empleador o sindicato	O	O
Seguro médico adquirido directamente de una compañía de seguros	O	O
Medicare (para personas con al menos 65 años de edad, o que padezcan determinadas discapacidades)	O	O
Medicaid, Asistencia Médica (o cualquier tipo del plan asistido por el gobierno para aquellas personas con bajos ingresos o discapacidad)	O	O
TRICARE (CHAMPUS) u otro servicio de salud militar	O	O
VA (incluidos aquellos que alguna vez han usado o se han inscrito en el cuidado de la salud de VA)	O	O
Seguro médico para nativos americanos	O	O
Cualquier otro tipo de seguro médico o plan de cobertura para su salud	O	O
No tengo seguro médico	O	O

*If they answered yes to Cualguier otro tipo de seugro

Usted escribió que actualmente está cubierto por otro tipo the seguro médico o plan de cobertura médico. Podría usted por favor especificar que es?

Si actualmente <u>no tiene</u> una cobertura médica, ¿aproximadamente cuándo fue la última vez que la tuvo?

Mes ☐

Año ☐

Los navegadores de seguros informan a los usuarios acerca de los planes de servicios médicos, los créditos fiscales y la distribución de los costes. Además, ayudan a las personas a inscribirse en planes de salud con prestaciones disponibles en los seguros médicos del mercado. ¿Alguna vez ha usado un navegador de seguros?

- ○ Sí
- ○ No

Un navegador para pacientes o un defensor de pacientes guía al paciente a través del sistema médico y lo ayuda a comunicarse con los proveedores sanitarios. El navegador para pacientes también funciona para superar los obstáculos que se interponen en el acceso del paciente a la recepción de asistencia médica. ¿Alguna vez ha usado un navegador para pacientes o un coordinador de cuidados para pacientes?

- ○ Sí
- ○ No

Telehealth ('tele-salud') es un término amplio que se refiere al suministro de educación sanitaria y servicios médicos a través telecomunicación. Incluye la monitorización remota de constantes vitales, consultas, evaluaciones, diagnósticos y prescripciones. ¿Alguna vez ha usado telehealth?

- ○ Sí
- ○ No

¿Tiene una persona que considere su médico personal o su proveedor de atención médica?

- Sí, solo una
- Sí, más de una
- No, no tengo una persona a la que considere mi médico personal o mi proveedor de atención médica.

¿Tiene en la actualidad alguna factura de servicios sanitarios que se están pagando pasado el plazo límite?

- Sí
- No

¿En algún momento en los últimos 12 meses ha necesitado una visita médica pero no pudo hacerla por el coste?

- Sí
- No

Aparte del coste, ¿en algún momento en los últimos 12 meses ha retrasado una visita médica por alguna de las siguientes razones?

	Sí	No
No pudo contactar por teléfono (línea ocupada, le pusieron en espera durante mucho tiempo, nadie respondió)	○	○
No pudo recibir una cita a tiempo	○	○
Una vez allí, tuvo que esperar mucho para ser atendido por un doctor	○	○
La clínica no estaba abierta cuando llegó	○	○
No tenía transporte	○	○
Tenía que cuidar a otro familiar necesitado (niños, ancianos, etc.)	○	○
No pudo tomarse el día en el trabajo	○	○
Tuvo problemas en la comunicación debido al idioma	○	○

Además de en los últimos 12 meses, ¿alguna vez tuvo que posponer recibir asistencia médica porque tenía preocupaciones acerca del coste?

- Sí
- No

Además de en los últimos 12 meses, ¿alguna vez tuvo que posponer recibir asistencia médica por la distancia o problemas con el transporte?

- Sí
- No

Sin incluir los medicamentos sin receta médica, ¿alguna vez en los últimos 12 meses no tomó su medicación como se le prescribió debido al coste?

- Sí
- No
- No se aplica (no le recetaron ningún medicamento)

¿Alguna vez tuvo dificultades para obtener una receta médica debido a que no tenía un medio de transporte fiable?

- Sí
- No
- No se aplica (no le recetaron ningún medicamento)

Una revisión rutinaria es un chequeo general físico, no un examen para una condición médica, enfermedad o lesión específica. ¿Cuánto tiempo ha pasado desde que tuvo una revisión rutinaria hecha por un médico u otro profesional de salud por última vez?

- Menos de 12 meses
- Entre uno y menos de dos años
- Entre 2 y menos de 3 años
- Entre 3 y menos de 5 años
- 5 años o más
- Nunca

En los últimos 12 meses, ¿cuántas veces ha visitado a un profesional de salud por…

Una condición crónica (como artritis, diabetes, enfermedad cardíaca cáncer o asma)? [ ] Number of visits

Una afección grave (como un ataque al corazón, un hueso roto, una lesión, fiebre súbita, dolores de pecho agudos, ataques de asma graves)? [ ] Number of visits

Salud mental o de comportamiento? [ ] Number of visits

En los últimos 12 meses, ¿cuántas citas ha tenido en relación a un cáncer (exámenes, diagnósticos, tratamientos, o seguimiento)?

[ ] Number of visits

En los últimos 12 meses, ¿cuántas veces ha estado en la sala de emergencias?

[ ] Number of visits

En los últimos 12 meses, ¿cuántas veces ha acompañado a otra persona a una cita médica o a una visita a un profesional de salud?

[ ] Number of visits

En los últimos 12 meses, ¿cuántas veces ha acompañado a otra persona a una cita relacionada con cáncer (exámenes, diagnósticos, tratamientos, o seguimiento)?

[ ] Number of visits

Incluyendo todo tipo de dentistas (higienista dental, dentista, ortodoncista, cirujano dental y cualquier otro tipo de especialista dental), ¿cuánto tiempo hace que vistió por última vez un dentista o una clínica dental por cualquier motivo?

- ○ Menos de 12 meses
- ○ Entre 1 y menos de 2 años
- ○ Entre 2 y menos de 3 años
- ○ Entre 3 y menos de 5 años
- ○ 5 años o más
- ○ Nunca

Lista de problemas

Por favor, indique si alguna circunstancia de las siguientes ha sido un problema para usted en los últimos 30 días.

	Sí	No
Cuidado de los niños	○	○
Cuidado de un adulto dependiente	○	○
Incapaz de trabajar debido a una discapacidad	○	○
Un niño con necesidades de educación especial insatisfechas	○	○
Jornadas laborales reducidas (incluyendo permisos o despidos)	○	○
Seguro médico	○	○
Prestaciones por desempleo	○	○
Prestaciones por discapacidad	○	○
SNAP/WIC (programas públicos de salud y nutrición)	○	○
Quedarse sin comida antes de poder comprar más	○	○
No se puede permitir por razones económicas una dieta equilibrada	○	○
Insectos o roedores en la vivienda	○	○
Pintura con plomo en la vivienda	○	○
Moho o humedades en la vivienda	○	○
Amenaza de desahucio	○	○

Servicios inestables	○	○
Limpieza general de la vivienda	○	○
Hacinamiento o saturación en la vivienda	○	○
Transporte	○	○
Cuidados médicos (para usted u otro miembro)	○	○
Estatus legal (preocupación por la situación de inmigración de la familia)	○	○
Estabilidad personal y familiar (por ejemplo, violencia doméstica, conflictos de tutela o custodia, bienestar de los niños)	○	○
Otro	○	○

*If they answer yes to Otro

Usted indicó que usted ha tenido otros problemas durante los pasados 30 días. Podría usted por favor especificar cuáles fueron esos problemas?

Por favor, indique si alguna de las siguientes circunstancias ha sido un problema para usted durante 2019.

	Sí	No
Cuidado de niños	○	○
Cuidado de un adulto dependiente	○	○
Incapaz de trabajar debido a una discapacidad	○	○
Un niño con necesidades de educación especial insatisfechas	○	○
Jornadas laborales reducidas (incluyendo permisos o despidos)	○	○
Seguro médico	○	○
Prestaciones por desempleo	○	○
Prestaciones por discapacidad	○	○
SNAP/WIC (programas públicos de salud y nutrición)	○	○
Quedarse sin comida antes de poder comprar más	○	○
No se puede permitir por razones económicas una dieta equilibrada	○	○
Insectos o roedores en la vivienda	○	○
Pintura con plomo en la vivienda	○	○
Moho o humedades en la vivienda	○	○
Amenaza de desahucio	○	○
Servicios inestables	○	○

Limpieza general de la vivienda	O	O
Hacinamiento o saturación en la vivienda	O	O
Transporte	O	O
Cuidados médicos (para usted u otro miembro)	O	O
Estatus legal (preocupación por la situación de inmigración de la familia)	O	O
Estabilidad personal y familiar (por ejemplo, violencia doméstica, conflictos de tutela o custodia, bienestar de los niños)	O	O
Otro	O	O

*If they answer yes to Otro

Usted indicó que usted ha tenido otros problemas durante el 2019. Podría usted por favor especificar cuáles fueron esos problemas?

[ ]

Acceso al teléfono y a la computadora

¿Posee o utiliza usted o algún miembro de su vivienda alguno de los siguientes tipos de computadoras?

	Sí	No
Computadora de mesa, computadora portátil, tablet u otro tipo de computadora inalámbrica?	O	O
Smartphone	O	O

¿Tiene usted o algún miembro de su vivienda acceso a internet?

- O Sí
- O No

*If they answer yes

¿Tiene usted o algún miembro de su vivienda acceso a internet usando alguno de los dispositivos siguientes?

	Sí	No
Plan de datos móviles para un smartphone u otro tipo de dispositivo móvil	○	○
Servicio de internet de banda ancha (alta velocidad) como cables, fibra óptica o servicio DSL instalado en su casa	○	○
Servicio de internet por satélite instalado en su casa	○	○
Servicio de internet por línea conmutada instalada en su casa	○	○

¿Cómo es la señal telefónica en su casa?

- ○ Buena
- ○ Suficiente
- ○ Mala

Posesión de vehículos

¿Cuántos vehículos posee usted o los miembros de su vivienda? Incluyendo carros, camiones, todoterrenos o furgonetas. *No incluya vehículos recreativos como motocicletas, tráilers o remolques, autocaravanas o casas sobre ruedas, ATV, motonieves, barcos o aviones.*

[ ] vehicles

¿Cuántos vehículos funcionan y están disponibles para el uso de los miembros en este momento

[ ] vehicles

¿Posee usted o alguno de los miembros de su vivienda alguna motocicleta?

- ○ Sí
- ○ No

*If they answer yes

¿Cuántas motocicletas funcionan y están disponibles para el uso de los miembros en este momento?

[ ] vehicles

Usted utiliza la moto principalmente para…

- ○ Ocio
- ○ Desplazarse al trabajo o la escuela
- ○ Hacer mandados/recados

Transporte

¿Tiene licencia válida para conducir?

- Sí
- No

¿Tiene alguien en su vivienda un carné o licencia para conducir válida?

- Sí
- No

¿Alguna vez ha dejado un trabajo o lo ha perdido porque no tenía un medio de transporte de confianza para llegar?

- Sí
- No

¿La falta de opciones de transporte le impide ir al trabajo ahora?

- Sí
- No

¿Cuál es la cantidad máxima que estaría dispuesto a pagar por un viaje de ida desde o hacia su lugar de trabajo?

- Menos de un dólar
- $ 1.00- $ 1.99
- $ 2.00- $ 2.99
- $ 3.00 o más

¿Cuál es la cantidad máxima que estaría dispuesto a pagar por un viaje de ida desde o hacia un servicio o cita médica?

- Menos de un dólar
- $ 1.00- $ 1.99
- $ 2.00- $ 2.99
- $ 3.00 o más

Si tiene que ir a algún sitio en carro, ¿qué dificultad tiene en llegar allí?

- Mucha dificultad
- Alguna dificultad
- Sin dificultad

El transporte médico no urgente (NEMT) es un servicio para los beneficiarios de Medicaid que cualifican por necesidades de transporte. Ofrece transporte para las citas no urgentes pero médicamente necesarias. ¿Alguna vez ha usado NEMT (para usted o para acompañar a alguien)?

- Sí
- No

*If they answer yes

Cuando utilizó NEMT, ¿experimentó alguna de las siguientes circunstancias?

	Sí	No
El vehículo llevaba a otros pasajeros al mismo tiempo	○	○
Hubo dificultad para programar un viaje (hacer una reserva)	○	○
El vehículo llegó tarde	○	○
El vehículo no apareció	○	○
El vehículo no pudo acomodar las necesidades físicas (como la accesibilidad para sillas de ruedas)	○	○
El recorrido tomó demasiado tiempo porque tuvo que hacer otras paradas	○	○

¿Utiliza alguno de los siguientes medios para llegar a sus citas médicas?

	Sí	No
Conduce usted	O	O
Le lleva algún miembro de su familia o un amigo	O	O
Camina (o va en silla de ruedas)	O	O
Va en bicicleta	O	O
Toma el transporte público (autobús, tren, metro)	O	O
Toma un taxi	O	O
Usa una plataforma de transporte compartido de pago como Uber o Lyft	O	O
Usa una furgoneta o un autobús de transporte proporcionado por su lugar de residencia	O	O
Usa una furgoneta o autobús lanzadera para personas mayores o personas con discapacidad	O	O
Usa transporte ofrecido por el centro médico (como un carro o furgoneta, o un transporte para no emergencias)	O	O
Otro	O	O

*If they answer yes to Otro

Usted indicó que usted usa otro tipo de transporte para movilizarse a sus citas de salud o citas médicas. Podría usted por favor especificar?

¿Hay algún pariente o amigo que normalmente transporte a uno de los miembros de su hogar?

- ○ Sí
- ○ No

Durante el último año, ¿tuvo algún problema con el transporte que le impidió hacer lo siguiente?

	Sí	No
Visitar a amigos o familiares que no viven con usted	○	○
Asistir a servicios religiosos	○	○
Participar en clubs, clases, u otras actividades organizadas	○	○
Realizar salidas de ocio (como salir a comer, apostar, escuchar música, ver una película, ir a un partido o un evento deportivo)	○	○
Solicitar asistencia médica	○	○

En los últimos 30 días, ¿con qué frecuencia utilizó alguno de los siguientes medios de transporte para llegar a destinos fuera de su vivienda?

	7 días a la semana	5-6 días a la semana	2-4 días a la semana	Una vez por semana o menos	Nunca
Condujo usted en un vehículo personal (carro, todoterreno, furgoneta, camioneta, moto)	○	○	○	○	○
Caminó (o fue en silla de ruedas)	○	○	○	○	○
Fue en bicicleta	○	○	○	○	○
Le transportó un miembro de su familia o un amigo	○	○	○	○	○
Tomó transporte público (autobús, tren, metro o subterráneo)	○	○	○	○	○
Tomó un taxi	○	○	○	○	○
Usó una plataforma de servicio compartido de pago como Uber o Lyft	○	○	○	○	○
Usó una furgoneta o un autobús de transporte proporcionado por su lugar de residencia	○	○	○	○	○
Usó una furgoneta o autobús lanzadera para personas mayores o personas discapacitadas	○	○	○	○	○
Condujo un carrito de golf, Segway, o un cortacésped.	○	○	○	○	○
Condujo maquinaria agrícola	○	○	○	○	○
Montó un caballo, burro o asno o condujo un carruaje tirado por caballos	○	○	○	○	○

Distancia de los trayectos

¿Actualmente, tiene usted trabajo?

- ○ Sí
- ○ No

*If they answer yes

¿Cuánta distancia debe recorrer normalmente de casa al trabajo (solo de ida)?

- ○ Menos de una milla
- ○ Entre 1 y menos de 5 millas
- ○ Entre 5 y menos de 10 millas
- ○ Entre 10 y menos de 15 millas
- ○ Entre 15 y menos de 30 millas
- ○ 30 o más millas

¿Cuánta distancia recorre de ida normalmente desde su casa por cada una de las siguientes razones?

	Menos de una milla	De una a menos de 5 millas	De 5 a menos de 10 millas	De 10 a menos de 15 millas	De 15 a menos de 30 millas	30 millas o más
Para comprar productos de necesidad básica para su hogar (comida, ropa, u otros productos domésticos)	○	○	○	○	○	○
Para servicios regulares (no urgentes) médicos, dentales, farmacéuticos, u otro tipo de servicios de salud.	○	○	○	○	○	○
Para recibir asistencia médica por una emergencia muy grave	○	○	○	○	○	○
Para recibir asistencia médica por una emergencia menos grave	○	○	○	○	○	○

De media, ¿cuánto tiempo le toma desplazarse (solo de ida) para citas médicas, dentales, u otro tipo de servicios de salud?

- Menos de 5 minutos
- De 5 a 9 minutos
- De 10 a 19 minutos
- De 20 a 29 minutos
- 30 minutos o más

En los últimos 6 meses, ¿cuántos viajes realizó para citas médicas (para usted o para otros) que estaban al menos a 50 millas de distancia (solo de ida) desde su casa?

____ trips

En los últimos 2 meses, ¿cuántos viajes realizó que fueran al menos de 50 millas (solo de ida) desde su casa (por cualquier motivo)?

____ trips

Demografía

¿En qué condado de Nebraska reside actualmente?

¿Cuál es el código postal de su residencia?

¿En qué año nació?

¿Es usted…?

- Hombre
- Mujer
-

¿Es usted de origen hispano (latino/a)?

- ○ Sí
- ○ No

¿Cuál es su raza? (seleccione todas las casillas que aplican)

- ☐ Blanco
- ☐ Negro / Afro-americano
- ☐ Nativo americano o nativo de Alaska
- ☐ Asiático / Isleño del Pacífico/ Nativo hawaiano

- ☐ Prefiero no contestar

¿Cuál es su estado civil? Marque solo UNA casilla.

- ○ Casado/a o viviendo con una pareja (cohabitando)
- ○ Divorciado/a o separado/a
- ○ Viudo/a
- ○ Soltero/a (nunca se ha casado, ni vive con una pareja)

¿Cuál es el nivel más alto de educación que usted ha completado?

- ○ Menos de graduado de la secundaria
- ○ Diploma de escuela secundaria o GED
- ○ Algún colegiado o grado técnico, vocacional, asociado, o grado de iniciación (2 años, LPN, etc.)
- ○ Grado universitario o superior (4 años, BA, BS, RN, máster(s), doctorado, Derecho, Medicina)

En la actualidad, usted… (seleccione todas las casillas que aplican)

- ☐ es amo/a de casa
- ☐ es estudiante
- ☐ está jubilado
- ☐ es empleado remunerado (a jornada completa o parcial)
- ☐ es autónomo
- ☐ lleva desempleado menos de un año
- ☐ lleva desempleado un año o más
- ☐ no puede trabajar por alguna discapacidad
- ☐ Otro

*If they answer Otro

Cuando se le preguntó que es usted actualmente, usted indicó otro. Podría usted por favor especificar?

____

¿Alguna vez ha servido en las Fuerzas Armadas estadounidenses, Reservas o en la Guardia Nacional?

- ○ Sí
- ○ No

¿Nació en los Estados Unidos?

- ◯ Sí
- ◯ No

*If they answer no

¿En qué año vino a vivir a los Estados Unidos?

[ ]

¿Qué lengua utiliza principalmente en su hogar?

- ◯ Inglés
- ◯ Español
- ◯ Otro

*If they answer Otro

Usted indicó que el idioma principal que se usa en su casa era otro. Podría usted por favor especificar?

Incluyéndose a usted, ¿cuántos miembros de su unidad doméstica tienen 19 años o más?

Adult(s)

Incluyéndose a usted, ¿cuántos adultos de 65 años o más viven en su hogar?

Adult(s)

¿Cuántas personas de 18 años o menos viven en su hogar?

Children

¿Cuál es el salario anual de su unidad doméstica (antes de impuestos), de todas las fuentes de ingresos, en este último año?

- De $0 a $9.999
- De $10.000 a $19.999
- De $20.000 a $29.999
- De $30.000 a $39.999
- De $40.000 a $49.999
- De $50.000 a $74.999
- $75.000 o más

Appendix B: Communications

Invitation - English

DATE

«City» Resident
«Addy1» «Addy2»
«City», «STATE_ABBR» «ZIP»-«ZIP4»

Dear «City» Resident,

I am writing to ask for your household's help with a survey called the Nebraska Health Care Access Survey. The questions on this survey will help provide information regarding Nebraskans' access to health care. This survey will help test the relationship between various measures and the impact they have on Nebraskans' health and access to health care.

This effort can only be successful with your help. We need **the adult (age 19 or over) from your household who has the next birthday after October 1, 2020** to do the survey.

To access this survey online, please go to the link listed below and enter your unique identification number.

Survey Link: https://go.unl.edu/health
Unique Identification Number:

This voluntary survey should take about 20 minutes. If you are unable to finish it in one sitting, you can click on the link again to pick up where you left off.

Your answers will be kept confidential and you can skip any questions you prefer not to answer. Any identifying information about you will be removed from the data before they are provided to ensure that no individual can be identified in the results. The data will only be used for evaluation and planning purposes. Your house was randomly selected from all Nebraska addresses.

If you have any questions about the survey, please do not hesitate to contact the Bureau of Sociological Research (BOSR) who is conducting this evaluation on behalf of UNMC, at (402) 472-3672 or bosr@unl.edu. You may also ask any questions concerning this project at any time by contacting me at kendra.ratnapradipa@unmc.edu. If you have questions about your rights as a research participant, you can call the UNMC Institutional Review Board at (402) 559-6463 and reference IRB # 605-20-EX.

We have enclosed a small gift of $1 to thank you for helping with the survey. Thank you for your help.

Sincerely,

Kendra L Ratnapradipa

Kendra L. Ratnapradipa, PhD
Principal Investigator
College of Public Health
University of Nebraska Medical Center

University of Nebraska Medical Center
College of Public Health | Department of Epidemiology
984395 Nebraska Medical Center | MCPH 3025 | Omaha, NE 68198-4395 | 402.552.7238 |
Kendra.ratnapradipa@unmc.edu | unmc.edu/publichealth

Invitation - Spanish

DATE

«City» Resident
«Addy1» «Addy2»
«City», «STATE_ABBR» «ZIP»-«ZIP4»

Estimado residente de «Ciudad»:

Le escribo para solicitar la ayuda de su familia con la encuesta denominada «Acceso a la atención médica de Nebraska». Las preguntas de esta encuesta ayudarán a obtener información sobre el acceso de los residentes de Nebraska a la asistencia de salud. Dicha encuesta contribuirá a examinar la relación entre diversas medidas y el impacto que tienen sobre la salud y el acceso a los servicios sanitarios de los residentes de Nebraska.

Este esfuerzo solo puede tener éxito con su ayuda. Necesitamos la participación **del adulto (mayor de 19 años) en su hogar cuyo próximo cumpleaños sea después del 1 de octubre de 2020.**

Para acceder a esta encuesta en línea, por favor vaya al enlace que se muestra a continuación e ingrese su número de identificación único.

Enlace a la encuesta: https://go.unl.edu/health
Número de identificación único:

La encuesta es voluntaria y se requieren alrededor de 20 minutos para completarla. Si por algún motivo usted no puede terminarla de una vez, puede regresar al enlace para retomarla desde donde la dejó.

Sus respuestas serán mantenidas en confidencialidad y usted puede optar por no contestar cualquier pregunta. Cualquier dato personal que pueda identificarlo será eliminado de nuestros registros antes que se utilice en la encuesta para asegurar el anonimato de los resultados. Los datos solo se utilizarán con fines evaluativos y de planificación. Su hogar ha sido aleatoriamente seleccionado entre todos los domicilios de Nebraska.

En caso de que usted tenga cualquier pregunta acerca de la encuesta, por favor no dude en contactar con la Oficina de Investigación Sociológica de UNL (BOSR), responsable por la conducción de esta evaluación de parte de UNMC, a través del teléfono (402) 472-3672 o por medio del correo electrónico bosr@unl.edu. Además, en cualquier momento usted puede solicitar explicaciones sobre el proyecto por medio del correo electrónico kendra.ratnapradipa@unmc.edu. Si usted tiene alguna duda sobre sus derechos como participante de esta investigación, llame al Comité de Revisión Institucional de UNMC a través del número (402) 559-6463 haciendo referencia al IRB # 605-20-EX.

Hemos añadido un pequeño obsequio de $1 para agradecerle su ayuda con la encuesta. Muchas gracias por su colaboración.

Atentamente,

Kendra L. Ratnapradipa

Kendra L. Ratnapradipa, PhD
Principal Investigator
College of Public Health
University of Nebraska Medical Center

University of Nebraska Medical Center
College of Public Health | Department of Epidemiology
984395 Nebraska Medical Center | MCPH 3025 | Omaha, NE 68198-4395 | 402.552.7238 |
Kendra.ratnapradipa@unmc.edu | unmc.edu/publichealth

Reminder - English

DATE

«City» Resident
«Addy1» «Addy2»
«City», «STATE_ABBR» «ZIP»-«ZIP4»

Dear «City» Resident,

We recently sent you a letter asking you to share your thoughts and experiences about health care in Nebraska and your access to it. Please do this if you haven't, however if you have, thank you. As a resident of Nebraska, you can provide valuable feedback that we cannot get anywhere else, and by doing so you will help improve the state of Nebraska for everyone.

This effort can only be successful with your help. We need **the adult (age 19 or over) from your household who has the next birthday after October 1, 2020** to do the survey.

To access this survey online, please go to the link listed below and enter your unique identification number.

> **Survey Link: https://go.unl.edu/health**
> **Unique Identification Number:**

This voluntary survey should take about 20 minutes. If you are unable to finish it in one sitting, you can click on the link again to pick up where you left off.

Your answers will be kept confidential and you can skip any questions you prefer not to answer. Any identifying information about you will be removed from the data before they are provided to ensure that no individual can be identified in the results. The data will only be used for evaluation and planning purposes. Your house was randomly selected from all Nebraska addresses.

If you have any questions about the survey, please do not hesitate to contact the Bureau of Sociological Research (BOSR) who is conducting this evaluation, on behalf of UNMC, at (402) 472-3672 or bosr@unl.edu. You may also ask any questions concerning this project at any time, by contacting me at kendra.ratnapradipa@unmc.edu. If you have questions about your rights as a research participant, you can call the UNMC Institutional Review Board at (402) 559-6463 and reference IRB # 605-20-EX.

Thank you for helping. Your feedback will help improve the state's health care system for current and future Nebraska residents.

Sincerely,

Kendra L. Ratnapradipa

Kendra L. Ratnapradipa, PhD
Principal Investigator
College of Public Health
University of Nebraska Medical Center

University of Nebraska Medical Center
College of Public Health | Department of Epidemiology
984395 Nebraska Medical Center | MCPH 3025 | Omaha, NE 68198-4395 | 402.552.7238 |
Kendra.ratnapradipa@unmc.edu | unmc.edu/publichealth

Reminder - Spanish

DATE

«City» Resident
«Addy1» «Addy2»
«City», «STATE_ABBR» «ZIP»-«ZIP4»

Estimado residente de «Ciudad»:

Hace algunas semanas enviamos una carta a su dirección solicitando que un miembro de su familia completara una encuesta online. Según nuestros registros, aún nos queda recibir la respuesta de su hogar. Le escribimos para darle una última oportunidad para aportar sus opiniones y sugerencias sobre sus experiencias ante el acceso y el uso del servicio sanitario de Nebraska. Solo a través de las voces de los ciudadanos de Nebraska como usted podemos mejorar el sistema sanitario para toda la población del estado.

Este esfuerzo solo puede tener éxito con su ayuda. Necesitamos la participación **del adulto (mayor de 19 años) en su hogar cuyo próximo cumpleaños sea después del 1 de octubre de 2020.**

Para acceder a esta encuesta en línea, por favor vaya al enlace que se muestra a continuación e ingrese su número de identificación único.

Enlace a la encuesta: https://go.unl.edu/health
Número de identificación único:

La encuesta es voluntaria y se requieren alrededor de 20 minutos para completarla. Si por algún motivo usted no puede terminarla de una vez, puede regresar al enlace para retomarla desde donde la dejó.

Sus respuestas serán mantenidas en confidencialidad y usted puede optar por no contestar cualquier pregunta. Cualquier dato personal que pueda identificarlo será eliminado de nuestros registros antes que se utilice en la encuesta para asegurar el anonimato de los resultados. Los datos solo se utilizarán con fines evaluativos y de planificación. Su hogar ha sido aleatoriamente seleccionado entre todos los domicilios de Nebraska.

En caso de que usted tenga cualquier pregunta acerca de la encuesta, por favor no dude en contactar con la Oficina de Investigación Sociológica de UNL (BOSR), responsable por la conducción de esta evaluación de parte de UNMC, a través del teléfono (402) 472-3672 o por medio del correo electrónico bosr@unl.edu. Además, en cualquier momento usted puede solicitar explicaciones sobre el proyecto por medio del correo electrónico kendra.ratnapradipa@unmc.edu. Si usted tiene alguna duda sobre sus derechos como participante de esta investigación, llame al Comité de Revisión Institucional de UNMC a través del número (402) 559-6463 haciendo referencia al IRB # 605-20-EX.

Gracias por su ayuda. Sus comentarios ayudarán a mejorar el sistema sanitario del estado para los actuales y futuros residentes de Nebraska.

Atentamente,

Kendra L Ratnapradipa

Kendra L. Ratnapradipa, PhD
Principal Investigator
College of Public Health
University of Nebraska Medical Center

University of Nebraska Medical Center
College of Public Health | Department of Epidemiology
984395 Nebraska Medical Center | MCPH 3025 | Omaha, NE 68198-4395 | 402.552.7238 |
Kendra.ratnapradipa@unmc.edu | unmc.edu/publichealth

Final Reminder - English

DATE

«City» Resident
«Addy1» «Addy2»
«City», «STATE_ABBR» «ZIP»-«ZIP4»

Dear «City» Resident,

A few weeks ago we sent a letter to your address asking a member of your household to go online and complete a survey. To the best of our knowledge, we have yet to receive your household's responses. We are writing to give you one last chance to provide your thoughts and opinions, looking at your experiences about health care in Nebraska and your access to it. It is only by hearing from Nebraskans like you that we can improve health care system for everyone in the state.

This effort can only be successful with your help. We need **the adult (age 19 or over) from your household who has the next birthday after October 1, 2020** to do the survey.

We have made the survey available online because it allows us to collect the information more quickly and to be more responsible with our research money. However, because some people don't use the internet and it is important that we hear from all households, we've also enclosed a paper copy and a postage-paid return envelope.

To access this survey online, please go to the link listed below and enter your unique identification number.

Survey Link: https://go.unl.edu/health
Unique Identification Number:

If you do not want to do the survey online, please complete and return the enclosed paper questionnaire.

Your answers will be kept confidential and you can skip any questions you prefer not to answer. Any identifying information about you will be removed from the data before they are provided to ensure that no individual can be identified in the results. Your house was randomly selected from all Nebraska addresses. If you have any questions about the survey, please do not hesitate to contact the UNL Bureau of Sociological Research (BOSR) at (402) 472-3672 or bosr@unl.edu. You may also ask any questions concerning this project at any time, by contacting me at kendra.ratnapradipa@unmc.edu. If you have questions about your rights as a research participant, you can call the UNMC Institutional Review Board at (402) 559-6463 and reference IRB # 605-20-EX.

Thank you for helping. Your feedback will help improve the state's health care system for current and future Nebraska residents.

Sincerely,

Kendra L Ratnapradipa

Kendra L. Ratnapradipa, PhD
Principal Investigator
College of Public Health
University of Nebraska Medical Center

University of Nebraska Medical Center
College of Public Health | Department of Epidemiology
984395 Nebraska Medical Center | MCPH 3025 | Omaha, NE 68198-4395 | 402.552.7238 |
Kendra.ratnapradipa@unmc.edu | unmc.edu/publichealth

Final Reminder - Spanish

DATE

«City» Resident
«Addy1» «Addy2»
«City», «STATE_ABBR» «ZIP»-«ZIP4»

Estimado residente de «Ciudad»:

Hace algunas semanas enviamos una carta a su dirección solicitando que un miembro de su familia completara una encuesta online. Según nuestros registros, aún nos queda recibir la respuesta de su hogar. Le escribimos para darle una última oportunidad para aportar sus opiniones y sugerencias sobre sus experiencias ante el acceso y el uso del servicio sanitario de Nebraska. Solo a través de las voces de los ciudadanos de Nebraska como usted podemos mejorar el sistema sanitario para toda la población del estado.

Este esfuerzo solo puede tener éxito con su ayuda. Necesitamos la participación **del adulto (mayor de 19 años) en su hogar cuyo próximo cumpleaños sea después del 1 de octubre de 2020.**

Hemos hecho disponible la encuesta online porque nos permite recoger información más rápidamente y de manera más económicamente eficiente con el presupuesto de la investigación. Sin embargo, ya que algunas personas no utilizan internet y es importante recibir la información de todas las familias, también hemos añadido una copia impresa y un sobre con el franqueo pagado.

Para acceder a esta encuesta en línea, por favor vaya al enlace que se muestra a continuación e ingrese su número de identificación único.

Enlace a la encuesta: https://go.unl.edu/health
Número de identificación único:

Si no desea participar de la encuesta en línea, por favor complete y reenvíe el cuestionario impreso que se encuentra adjunto.

Sus respuestas serán mantenidas en confidencialidad y usted puede optar por no contestar cualquier pregunta. Cualquier dato personal que pueda identificarlo será eliminado de nuestros registros antes que se utilice en la encuesta para asegurar el anonimato de los resultados. Su hogar ha sido aleatoriamente seleccionado entre todos los domicilios de Nebraska. En caso de que usted tenga cualquier pregunta acerca de la encuesta, por favor no dude en contactarme por medio del correo electrónico kendra.ratnapradipa@unmc.edu. Si usted tiene alguna duda sobre sus derechos como participante de esta investigación, llame al Comité de Revisión Institucional de UNMC a través del número (402) 559-6463 haciendo referencia al IRB # 605-20-EX.

Gracias por su colaboración. Sus comentarios ayudarán a mejorar el sistema de salud del estado para los actuales y futuros residentes de Nebraska.

Atentamente,

Kendra L Ratnapradipa

Kendra L. Ratnapradipa, PhD
Principal Investigator
College of Public Health
University of Nebraska Medical Center

University of Nebraska Medical Center
College of Public Health | Department of Epidemiology
984395 Nebraska Medical Center | MCPH 3025 | Omaha, NE 68198-4395 | 402.552.7238 |
Kendra.ratnapradipa@unmc.edu | unmc.edu/publichealth

Additional Mailing

DATE

«City» Resident
«Addy1» «Addy2»
«City», «STATE_ABBR» «ZIP»-«ZIP4»

Dear «City» Resident,

About a month ago, we sent a letter to your address asking a member of your household to complete a survey discussing the health care system in Nebraska. We are worried that selected households might not think this survey is for them. As a resident of Nebraska, we value your thoughts and opinions about your experiences. These experiences could be your **access** to health care or it could be your **lack of access** to it. It could vary from your modes of transportation to appointments or your ability to connect a provider through the telephone or internet. It is only by hearing from Nebraskans in all parts of the state that we can improve the health care system as a whole. **Whether or not you actively use Nebraska's health care system, we want to hear from you.** We need the adult (age 19 or over) from your household who has the next birthday after October 1, 2020 to do the survey.

We have made the survey available online because it allows us to collect the information more quickly. However, because some people do not use the internet and it is important that we hear from **all households**, we have also enclosed a paper copy and a postage-paid return envelope.

To access this survey online, please go to the link listed below and enter your unique identification number.

Survey Link:
Unique Identification Number:

If you do not want to do the survey online, please complete and return the enclosed paper questionnaire.

Your answers will be kept confidential and you can skip any questions you prefer not to answer. Any identifying information about you will be removed from the data before they are provided to ensure that no individual can be identified in the results. Your house was randomly selected from all Nebraska addresses. If you have any questions about the survey, please do not hesitate to contact the UNL Bureau of Sociological Research (BOSR) at (402) 472-3672 or bosr@unl.edu. You may also ask any questions concerning this project at any time, by contacting me at kendra.ratnapradipa@unmc.edu. If you have questions about your rights as a research participant, you can call the UNMC Institutional Review Board at (402) 559-6463 and reference IRB # 605-20-EX.

Thank you for considering our request. Your feedback will help improve the state's health care system for all of Nebraska's residents.

Sincerely,

Kendra L Ratnapradipa

Kendra L. Ratnapradipa, PhD
Principal Investigator
College of Public Health
University of Nebraska Medical Center

University of Nebraska Medical Center
College of Public Health | Department of Epidemiology
984395 Nebraska Medical Center | MCPH 3025 | Omaha, NE 68198-4395 | 402.552.7238 |
Kendra.ratnapradipa@unmc.edu | unmc.edu/publichealth

Appendix C: Strata

Stratum 1: Urban large counties without oversample

Cass
Douglas
Lancaster
Sarpy
Saunders
Seward
Washington

Stratum 2: Urban small counties

Adams
Buffalo
Dakota
Dawson
Dixon
Dodge
Gage
Hall
Hamilton
Howard
Lincoln
Madison
Merrick
Platte
Scotts Bluff

Stratum 3: Rural counties

Antelope
Arthur
Banner
Blaine
Boone
Box Butte
Boyd
Brown
Burt
Cedar
Chase
Cherry
Cheyenne
Clay
Colfax
Cuming
Custer
Dawes
Deuel
Dundy
Fillmore
Franklin
Frontier
Furnas
Garden
Garfield
Gosper
Grant
Greeley
Harlan
Hayes
Hitchcock
Holt
Hooker
Jefferson
Johnson
Kearney
Keith
Keya Paha
Kimball
Knox
Logan
Loup
McPherson
Morrill
Nance
Nemaha
Nuckolls
Otoe
Pawnee
Perkins
Phelps
Pierce
Polk
Red Willow
Richardson
Rock
Saline
Sheridan
Sherman
Sioux
Stanton
Thayer
Thomas
Thurston
Valley
Wayne
Webster
Wheeler
York

Stratum 4: Oversample census tracts where at least 30% of the population is African American

31055000300
31055000600
31055000700
31055000800
31055001100
31055001200
31055005100
31055005200
31055005300
31055005400
31055005800
31055005901
31055005902
31055006000

31055006101
31055006102
31055006202
31055006301
31055006302
31055006303
31055006506

Stratum 5: Oversample census tracts where at least 30% of the population is Native American

31173940100
31173940200

Stratum 6: Oversample census tracts where at least 30% of the population is Hispanic

964800
010100
968400
968500
002000
002400
002600
002700
002800
002900
003000
003200
003300
003900
000200
000300
953700

Appendix D: Estimate of Sampling Error

The Health and Health Care Access in Nebraska sample is a stratified random sample of households in the state. Because the data were weighted to account for within household selection and population characteristics, the estimates of the sampling error are not straightforward. Table 4 presents margins of sampling error for some of the most likely sample sizes *not* taking the design effect from weighting into account. Exact margins of error for alternative specifications of sample size and reported percentages can be easily computed by using the following formula for the 95% confidence level:

Margin of error = 1.96 * square root (p(1-p)/n)

p = the expected proportion selecting the answer

n = number of responses

Table 4. Approximate Margins of Error of Percentages by Selected Sample Size <u>NOT</u> Accounting for Design Effect (Expressed In Percentages)*

Reported Percentage	Full Sample* n=1101	75% Sample n=825	50% Sample n=550	33.3% Sample n=367	25% Sample n=275	10% Sample n=110
50	2.95%	3.41%	4.18%	5.12%	5.91%	9.34%
40 or 60	2.89%	3.34%	4.09%	5.01%	5.79%	9.16%
30 or 70	2.71%	3.13%	3.83%	4.69%	5.42%	8.56%
20 or 80	2.36%	2.73%	3.34%	4.09%	4.73%	7.48%
10 or 90	1.77%	2.05%	2.51%	3.07%	3.55%	5.61%
5 or 95	1.29%	1.49%	1.82%	2.23%	2.58%	4.07%

When accounting for design effects due to weighting, the adjusted sampling error will be increased as is shown when comparing Table 4 to Table 5 where the design effect is incorporated:

Margin of error = square root (deff) * 1.96 * square root (p(1-p)/n)

deff = design effects

p = the expected proportion selecting the answer

n = number of responses

Table 5. Approximate Margins of Error of Percentages by Selected Sample Size Accounting for the Design Effect of Weighting (Expressed In Percentages)*

Reported Percentage	Full Sample* n=1101	75% Sample n=825	50% Sample n=550	33.3% Sample n=367	25% Sample n=275	10% Sample n=110
50	5.44%	6.29%	7.70%	9.43%	10.89%	17.22%
40 or 60	5.33%	6.16%	7.54%	9.23%	10.67%	16.87%
30 or 70	4.99%	5.76%	7.06%	8.64%	9.98%	15.78%
20 or 80	4.35%	5.03%	6.16%	7.54%	8.71%	13.77%
10 or 90	3.27%	3.77%	4.62%	5.66%	6.53%	10.33%
5 or 95	2.37%	2.74%	3.36%	4.11%	4.75%	7.50%

** 95% confidence interval states that in 95 out of 100 samples drawn using the same sample size and design, the interval will contain the population value.*

Appendix E: AAPOR Transparency Initiative Immediate Disclosure Items

1. Who sponsored the research study.
   Introduction
2. Who conducted the research study.
   Introduction
3. If who conducted the study is different from the sponsor, the original sources of funding will also be disclosed.
   Introduction
4. The exact wording and presentation of questions and response options whose results are reported. This includes preceding interviewer or respondent instructions and any preceding questions that might reasonably be expected to influence responses to the reported results.
   Appendix A
5. A definition of the population under study and its geographic location.
   Sampling Design
6. Dates of data collection.
   Data Collection Process and Response Rate
7. A description of the sampling frame(s) and its coverage of the target population, including mention of any segment of the target population that is not covered by the design. This may include, for example, exclusion of Alaska and Hawaii in U.S. surveys; exclusion of specific provinces or rural areas in international surveys; and exclusion of non-panel members in panel surveys. If possible the estimated size of non-covered segments will be provided. If a size estimate cannot be provided, this will be explained. If no frame or list was utilized, this will be indicated.
   Sampling Design
8. The name of the sample supplier, if the sampling frame and/or the sample itself was provided by a third party.
   Sampling Design
9. The methods used to recruit the panel or participants, if the sample was drawn from a pre-recruited panel or pool of respondents.
   Not applicable
10. A description of the sample design, giving a clear indication of the method by which the respondents were selected, recruited, intercepted or otherwise contacted or encountered, along with any eligibility requirements and/or oversampling. If quotas were used, the variables defining the quotas will be reported. If a within-household selection procedure was used, this will be described. The description of the sampling frame and sample design will include sufficient detail to determine whether the respondents were selected using probability or non-probability methods.
    Sampling Design
11. Method(s) and mode(s) used to administer the survey (e.g., CATI, CAPI, ACASI, IVR, mail survey, web survey) and the language(s) offered.
    Sampling Design and Questionnaire Design

12. Sample sizes (by sampling frame if more than one was used) and a discussion of the precision of the findings. For probability samples, the estimates of sampling error will be reported, and the discussion will state whether or not the reported margins of sampling error or statistical analyses have been adjusted for the design effect due to weighting, clustering, or other factors. Disclosure requirements for non-probability samples are different because the precision of estimates from such samples is a model-based measure (rather than the average deviation from the population value over all possible samples). Reports of non-probability samples will only provide measures of precision if they are accompanied by a detailed description of how the underlying model was specified, its assumptions validated and the measure(s) calculated. To avoid confusion, it is best to avoid using the term "margin of error" or "margin of sampling error" in conjunction with non-probability samples.

    Sampling Design, Design Effects, Appendix D

13. A description of how the weights were calculated, including the variables used and the sources of weighting parameters, if weighted estimates are reported.

    Data weights

14. If the results reported are based on multiple samples or multiple modes, the preceding items will be disclosed for each.

    Not applicable

15. Contact for obtaining more information about the study.

    Questions
